# Remodeling of bronchial epithelium caused by asthmatic inflammation affects its response to rhinovirus infection

**DOI:** 10.1038/s41598-021-92252-6

**Published:** 2021-06-17

**Authors:** Bogdan Jakiela, Ana Rebane, Jerzy Soja, Stanislawa Bazan-Socha, Anet Laanesoo, Hanna Plutecka, Marcin Surmiak, Marek Sanak, Krzysztof Sladek, Grazyna Bochenek

**Affiliations:** 1 Department of Internal Medicine, Faculty of Medicine, Jagiellonian University Medical College, Skawinska 8, 31-066 Kraków, Poland; 2 Institute of Biomedicine and Translational Medicine, University of Tartu, Tartu, Estonia

**Keywords:** Respiratory tract diseases, Infection, Innate immunity

## Abstract

Human rhinoviruses (HRV) are frequent cause of asthma exacerbations, however the influence of airway inflammation on the severity of viral infection is poorly understood. Here, we investigated how cytokine-induced remodeling of airway epithelium modulates antiviral response. We analyzed gene expression response in in vitro differentiated bronchial epithelium exposed to cytokines and next infected with HRV16. IL-13-induced mucous cell metaplasia (MCM) was associated with impaired ciliogenesis and induction of antiviral genes, resulting in lower susceptibility to HRV. Epithelial-mesenchymal transition caused by TGF-β was associated with increased virus replication and boosted innate response. Moreover, HRV infection per se caused transient upregulation of MCM markers and growth factors, followed by low-level virus replication and shedding. Our data suggest that the outcome of HRV infection depends on the type of lower airway inflammation and the extent of epithelial damage. Type-2 inflammation (eosinophilic asthma) may induce antiviral state of epithelium and decrease virus sensitivity, while growth factor exposure during epithelial repair may facilitate virus replication and inflammatory response. Additionally, responses to HRV were similar in cells obtained from asthma patients and control subjects, which implicates that antiviral mechanisms are not intrinsically impaired in asthma, but may develop in the presence of uncontrolled airway inflammation.

## Introduction

Asthma is a chronic inflammatory disease of the airways, characterized by reversible airway obstruction and hyperresponsiveness, with episodic worsening of symptoms, often related to respiratory tract infections or exposure to allergens^1^. Although the mechanism of asthma is not fully elucidated, approximately half of the patients show airway eosinophilia developing on type-2 (T2) immune background, while others with pauci-granulocytic or neutrophilic inflammation are generally classified as non-T2 subtype^2, 3^. Such a distinction was proposed based on the study analyzing the relationship between the type of airway inflammation and gene expression profile in bronchial epithelial cells^4^. Being the frontline between the host and environment, the bronchial epithelium is continuously exposed to respiratory pathogens, allergens, and air pollutants that stimulate innate immune responses but also induce tissue injury^5^. Repairing epithelial cells generate growth factors, e.g., transforming growth factor-β (TGF-β), which are essential for the proper restoring of epithelial integrity. At the same time, they trigger pro-fibrotic phenotype and epithelial-mesenchymal transition (EMT), thus contributing to airway remodeling in asthma^6^. Mediators secreted by inflammatory cells may modify those processes, altering the epithelial phenotype itself. An example of such a change is mucous cell metaplasia (MCM), a type of epithelial remodeling commonly seen in asthma, characterized by an increase in goblet cell number usually induced by chronic exposure to T2-cytokines (e.g., IL-13)^7, 8^. The structure and functions of the bronchial epithelium are thus compromised in asthma, which is believed to be the main reason for more severe responses to environmental triggers.

Infections with human rhinoviruses (HRV) are responsible for up to 90% of wheezing episodes in children, and 50 to 80% of asthma exacerbations in adults^9^. Nevertheless, repeated testing for respiratory pathogens revealed that asymptomatic HRV infections are ubiquitous in children and adult asthmatics^10, 11^. This indicates that certain host factors may influence the airway response to the virus, not always leading to the exacerbation of the disease. The HRV genus is highly diverse, with ~ 170 relatively stable lineages classified into three species A, B, and C^12^. They infect airway epithelial cells in both the upper and lower respiratory tract, with the majority of genotypes (most of HRV-A, including HRV16, and all HRV-B) utilizing intercellular adhesion molecule-1 (ICAM-1) as an entry receptor^13^. Sensing of viral dsRNA, transiently produced in the infected cell, leads to the production of type I and III interferons (IFN) and proinflammatory cytokines^14, 15^. IFN signaling results in a downstream expression of antiviral effector proteins called IFN-stimulated genes (ISGs) which act synergistically by inhibiting virus replication and mounting an ‘antiviral state’ in the host and surrounding cells^16^. This complex system of innate defense is critical for limiting the infection of airway epithelium. However, the question remains whether it is equally potent in the tissue damaged or remodeled by inflammatory cytokines?

We have recently reported that MCM induced by T2-cytokines decreased the susceptibility of bronchial epithelium to HRV infection^17^. It may be related to the reduced number of ciliated cells, which are the primary target for HRV in the intact airway epithelium, as demonstrated by our group^17^ and further confirmed by others^18–21^. Nevertheless, the reason for the lower vulnerability of goblet cells of MCM epithelium to HRV has not been explained so far. Likewise, the impact of non-T2 inflammatory conditions, e.g., mediated by IL-17A^22, 23^, on the response of infected epithelium has not been investigated in detail. An earlier report demonstrated synergy between IL-17A stimulation and response to HRV infection in primary human bronchial epithelial cells (HBECs)^24^, however, it was not verified in a polarized epithelium. Little is also known how exposure of mucociliary epithelium to TGF-β modulates the viral response, although the relatively high sensitivity of primary HBECs to HRV suggests that regenerating cells could be an easy target for the virus.

Based on that background, we hypothesized that the vulnerability of airway epithelium to HRV depends on the type and extent of remodeling induced by inflammatory conditions. To test that hypothesis, we analyzed the response to HRV16 infection in the bronchial epithelium differentiated in vitro and stimulated with cytokines to reproduce the structural changes associated with asthma, such as IL-13-induced MCM and TGF-β-induced EMT. We investigated expression of antiviral genes, particularly IFN-stimulated antiviral effectors, and subsequent cellular response to infection. We also checked if these processes are differentially regulated in cells derived from asthma patients with different inflammatory patterns in the lower airways.

## Results

### Increased baseline expression of antiviral genes in the epithelium with IL-13-induced mucous cell metaplasia

**Figure 1:**
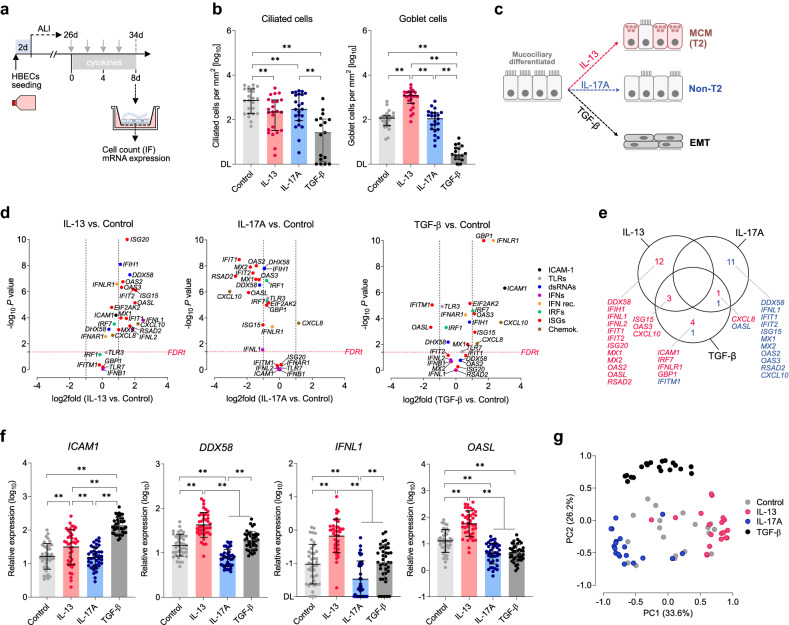
Antiviral gene expression profile in bronchial epithelium with cytokine-induced remodeling. (**a**) Bronchial epithelial cells were differentiated in an air–liquid interface (ALI) system for 26 d and next cultured in the presence of IL-13, IL-17A, or TGF-β (or control) for an additional 8 days. (**b**) The number of ciliated (Tubulin-βIV +) and goblet (Mucin-5AC +) cells in different culture conditions. Data are shown as medians and quartile range (n = 23 [n = 17 in case of TGF-β]). Friedman’s rank test: **P < 0.01. *DL* detection limit (~ 1 cell per mm^2^). (**c**) Schematic representation of the three types of airway epithelial remodeling analyzed in this study. *MCM* mucous cell metaplasia, *T2* type-2 inflammation, *EMT* epithelial mesenchymal transition. (**d**) Relative expression changes of viral response genes in ALI-epithelium cultured in the presence of indicated cytokines compared to untreated control (n = 19, 2-sided paired t-test *P* < 0.05, FDRt *q* = 0.05). *TLRs* toll-like receptors, *IFNs* interferons, *IFN rec.* receptors for IFNs, *IRFs* IFN regulatory factors, *ISGs* IFN-stimulated genes. (**e**) Venn diagram summarizing differences in viral response gene expression in different culture conditions, only targets significantly (n = 19, *P* < 0.05, FDRt *q* = 0.05) upregulated (log2fold > 1, *red*) or downregulated (log2fold < 1, *navy*) are shown. (**f**) Relative expression of *ICAM1*, *DDX58*, *IFNL1*, and *OASL* in airway epithelium cultured as in ‘a’. Horizontal bars represent means and SD (n = 40). RM 1-way ANOVA (Tukey): **P < 0.01. (**g**) Principal component (PC) analysis of viral response genes (n = 19).

To investigate how asthmatic inflammation affects epithelial structure and antiviral responses, we introduced an in vitro model of cytokine-induced remodeling using HBECs isolated from airway biopsies sampled in asthma patients and control subjects (n = 40; Supplementary Table S1 and Fig. S1). HBECs were mucociliary differentiated at the air–liquid interface (ALI) and next chronically exposed to IL-13, IL-17A or TGF-β (Fig. 1a). Incubation with IL-13 resulted in MCM, reflected by an increased number (~ ninefold) of goblet cells (Fig. 1b), and a distinctive mRNA expression profile with upregulation of *MUC5AC* and related T2-markers (e.g., *CLCA1*; Supplementary Fig. S2a–d). In turn, TGF-β_1_ led to a profound change in the epithelial structure, including almost the entire loss of differentiated apical cells (Fig. 1b) and a gene expression profile representative of EMT, including upregulation of Snail-family transcription factors (e.g., *SNAI1*) and extracellular matrix proteins. Interestingly, incubation with IL-17A resulted in a decrease (~ twofold) in the number of ciliated cells, with only minor changes in mRNA expression, demonstrated by the co-clustering of IL-17A and control gene expression profiles (Supplementary Fig. S2e,f). This cell culture model enabled the reproduction of the major structural changes of the bronchial epithelium found in asthma (Fig. 1c).

Next, we tested how cytokine-induced remodeling affects the ‘antiviral state’ of the bronchial epithelium. We investigated the mRNA expression of several genes involved in the viral response, i.e., dsRNA sensors (e.g., *DDX58* encoding RIG-1 [retinoic acid-inducible gene 1]), IRFs (IFN-regulatory factors), type I and III IFNs (e.g., *IFNL1* encoding IL-29) and ISGs (all listed in Supplementary Table S2). Within the latter group, we included major antiviral effectors, such as *RSAD2* (Viperin; interferes with virus replication), OAS-family (2’-5’-oligoadenylate synthase; participate in the degradation of viral RNA), *IFIT1 * and *IFIT2* (inhibit viral RNA translation), *MX1* and *MX2* (inhibit viral particle assembly), and *ISG15* (introduces protein modifications interfering with virus cycle)^25, 26^. As it turned out, IL-13-induced MCM resulted in a marked upregulation of several genes linked to the viral response (Fig. 1d,e), primarily dsRNA sensors (e.g., *DDX58*, mean 3.2-fold), type III IFNs (*IFNL1*, sixfold; *IFNL2*, 3.6-fold), and the majority of ISGs (e.g., fourfold for *OASL* and *ISG20*) (Fig. 1f). mRNA expression of *ICAM1* was higher (1.8-fold) in the epithelium with IL-13-induced MCM compared to control conditions (Fig. 1f).

Exposure of the bronchial epithelium to IL-17A resulted in a significant decrease in the expression of most ISGs, dsRNA sensors, and IRFs (Fig. 1d–f). As expected, IL-17A treatment led to a substantial induction of *CXCL8* (IL-8; mean threefold). Interestingly, TGF-β-induced EMT had more complex effects on the viral response pathway, including strong upregulation of *ICAM1* (8.3-fold), *IFNLR1* (a subunit of type III IFN receptor, fivefold), and selected ISGs (e.g., *ISG15*, *OAS3* and *GBP1*), albeit ~ threefold downregulation of *IFITM1* and *OASL* (Fig. 1d,e). These results were confirmed using unsupervised methods; for example, the principal component analysis showed three distinct gene expression patterns reflecting the three cytokine conditions (Fig. 1g).

### Cytokine-induced structural changes of bronchial epithelium influence sensitivity to HRV infection

**Figure 2:**
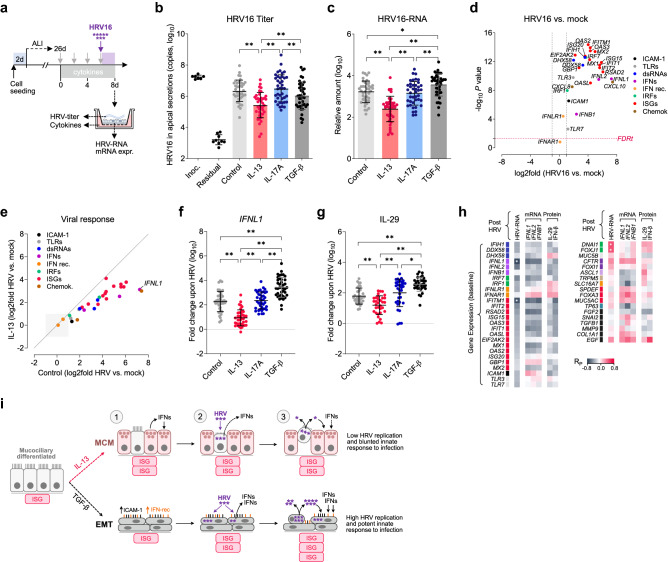
Reduced susceptibility to HRV16 infection in bronchial epithelium with IL-13-induced mucous cell metaplasia (MCM). (**a**) Air–liquid interface (ALI) differentiated bronchial epithelium was cultured with IL-13, IL-17A, or TGF-β (or w/o cytokines) and then infected 48 h with HRV16. (**b**) HRV16 titer in apical secretions in the indicated conditions, the inoculum (inoc.), and after wash (residual). (**c**) Expression of HRV16-RNA in cell lysates. (**d**) Relative expression of antiviral genes, including toll-like receptors (TLRs), dsRNA sensors, interferons (IFNs), and interferon-stimulated genes (ISGs) in the HRV16-infected mucociliary epithelium (control conditions) compared to mock (n = 19, 2-sided paired t-test *P* < 0.05, FDRt *q* = 0.05). (**e**) Fold differences (HRV16 vs. mock) in the expression of antiviral genes in bronchial epithelium exposed to IL-13 or in control conditions. (**f**) Fold change in the expression of *IFNL1* mRNA, and (**g**) in the level of IL-29 in cell culture supernatant upon HRV16 infection in different conditions. Statistics (‘b’, ‘c’, ‘f’ and ‘g’): Bars represent means and SD (n = 40). RM 1-way ANOVA (Tukey): *P < 0.05, **P < 0.01. (**h**) Correlation heat map (Pearson’s coefficients [R_P_]; control conditions) showing the association between baseline mRNA expression of viral response (*left*) or structural (*right*) genes, and subsequent response to HRV16 (e.g., HRV-RNA and type III IFNs). n = 19, *P < 0.01. (**i**) A model of putative mechanism of HRV infection in remodeled bronchial epithelium. (1) The exposure of bronchial epithelium to IL-13 induces MCM, while stimulation with TGF-β leads to epithelial-mesenchymal transition (EMT). (2) MCM renders the epithelium less sensitive to infection, as HRV targets primarily sparsely distributed ciliated cells and does not efficiently replicate in mucous cells due to their ‘antiviral state’, while epithelium with EMT is more permissive to HRV infection. (3) The magnitude of innate inflammatory response is determined by HRV replication rate and autocrine action of type I and III IFNs.

Next, we checked the sensitivity of remodeled epithelium to HRV infection. For that, ALI-cultures were exposed to cytokines (or left untreated), and then infected with HRV16 for 48 h to assess virus replication and antiviral response (Fig. 2a). In the epithelium with IL-13-induced MCM, both HRV16 titers in apical secretions and HRV16-RNA in cell lysates were significantly reduced (mean ~ eightfold) compared to other culture conditions (Fig. 2b,c). There was no difference in HRV16 replication and shedding in IL-17A conditions compared to epithelium cultured without cytokines. In contrast, HRV16-RNA was significantly increased (~ twofold) in the epithelium with TGF-β-induced EMT, although the apical release was similar to that observed in control replicates (Fig. 2b,c).

As expected, HRV16 infection of epithelium differentiated in control conditions resulted in a marked induction of IFNs (mean 200-fold for *IFNL1*), and most of the analyzed antiviral effectors (Fig. 2d) with ISGs being the top group upregulated (10 to 100-fold). However, the induction of antiviral genes was significantly weaker in the epithelium with IL-13-induced MCM (Fig. 2e). For example, both the rise in *IFNL1* mRNA and IL-29 level were decreased in the presence of IL-13 compared to other conditions (Fig. 2f,g). Moreover, the sensitivity to HRV depended on the advancement of structural lesions, as only prolonged IL-13 exposure (> 4 d) and higher cytokine concentrations resulted in decreased virus replication and IFN-response (Supplementary Fig. S3). Nevertheless, a positive correlation between HRV16-RNA and IFN expression (Supplementary Fig. S4) suggests that the blunted response in MCM-epithelium is likely a derivative of decreased HRV replication, but not a lower potential of infected cells to induce IFNs. The innate response to HRV16 infection was comparable in IL-17A-treated and control cells (Supplementary Fig. S5). In contrast, the magnitude of the antiviral response was strongly enhanced after infection of epithelium with TGF-β-induced EMT, as the expression of most antiviral genes was ~ tenfold higher than in all other conditions (Fig. 2f,g; Supplementary Fig. S5).

In the search for factors influencing sensitivity to the virus, we performed a correlation analysis comparing baseline mRNA expression with the magnitude of post-infection response. As it turned out, both the rate of HRV16 replication and the associated IFN-response correlated negatively with baseline expression of type III IFNs and ISGs (e.g., *IFNL1* R = − 0.66, Fig. 2h). Additionally, HRV16 replication was positively associated with ciliogenesis markers (e.g., *DNAI1* R = 0.57, Fig. 2h). Similar results were obtained in the analysis comprising cytokine-treated cells (Supplementary Fig. S6a), although an additional inverse association between baseline expression of MCM markers and HRV replication (e.g., *SPDEF* R = − 0.53 for the whole dataset) was also observed. Furthermore, we noticed a characteristic biphasic pattern (Supplementary Fig. S6b), as extensive replication of HRV16 occurred either in cultures with a high cilia signature or in those with low expression of apical cell markers (i.e., less well-differentiated or upon exposure to TGF-β). Altogether, our data suggest that the sensitivity of bronchial epithelium to HRV likely depends on the inflammatory environment and the advancement of structural remodeling, such that IL-13-induced MCM protects against severe infection, while growth-factor induced EMT may facilitate virus replication and enhance inflammatory response (as summarized in Fig. 2i).

### HRV infection of the mucociliary epithelium is associated with a transient upregulation of mucous cell markers and growth factors

**Figure 3:**
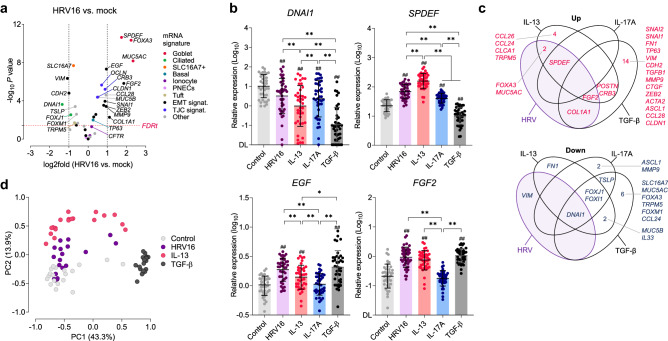
HRV16 infection modulates the expression of genes associated with remodeling of the bronchial epithelium. (**a**) Relative expression changes in structural and EMT-related genes in ALI-grown bronchial epithelium (32 days) infected with HRV16 (48 h). Vertical dashed lines indicate log2fold -1 or 1 (n = 19; 2-sided t-test *P* < 0.05 at FDRt *q* = 0.05). (**b**) Relative expression of *DNAI1*, *SPDEF*, *EGF*, and *FGF2* in HRV16-infected mucociliary epithelium compared to uninfected cells cultured in different conditions. Data are shown as means and SD (n = 40). RM 1-way ANOVA (Tukey): *P < 0.05, **P < 0.01. *DL* detection limit. (**c**) Venn diagrams showing changes in mRNA expression upon HRV16 infection and cytokine treatment. Only genes significantly (log2fold < − 1 or > 1, *P* < 0.05) up- (*red*) or downregulated (*navy*) when compared to uninfected control conditions are shown. (**d**) Principal component analysis of genes associated with remodeling in HRV16-infected or cytokine treated epithelium (IL-17A dataset not shown for clarity).

In the next part of the study, we examined whether HRV infection by itself could induce remodeling of the bronchial epithelium, and if such changes could be long-lasting. As expected, HRV16 infection of the mucociliary epithelium resulted in a significant decrease in the expression of cilia-associated genes (e.g., *DNAI1*, Fig. 3a), likely due to preferential targeting of ciliated cells by HRV and related damage of the mucociliary apparatus^17, 19, 20^. Additionally, we observed a strong (mean ~ fourfold) upregulation of all goblet cell markers studied (*SPDEF*, *FOXA3* and *MUC5AC*). The effect of HRV16 infection on epithelial gene expression was in many ways similar to that observed during IL-13-induced MCM (Fig. 3b,c), which was confirmed by multivariate analysis (Fig. 3d). HRV16 infection also led to a significant increase in expression of genes involved in EMT (e.g., *COL1A1*, *MMP9*, *SNAI1*, and *ZEB2*; Supplementary Fig. S7) and growth factors (e.g., ~ fourfold for *EGF* and *FGF2*, and to a lesser extent *TGFB1*).

**Figure 4:**
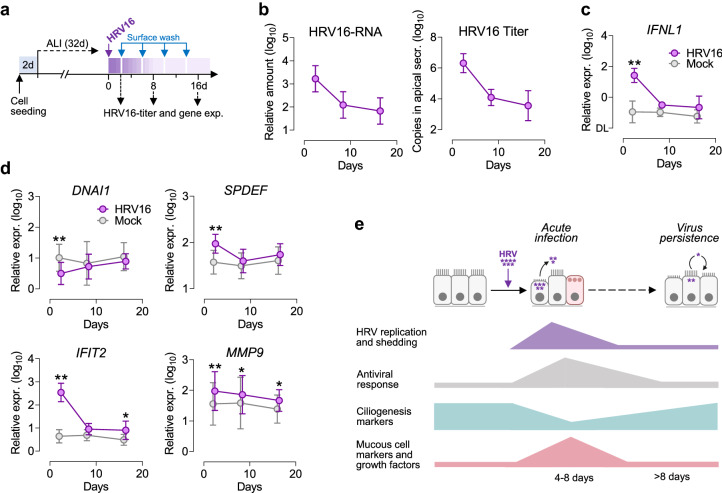
Prolonged HRV16 infection of in vitro differentiated bronchial epithelium. (**a**) Model of prolonged HRV infection. Air–liquid interface (ALI)-grown bronchial epithelium was apically infected with HRV16 and next incubated for 16 days with surface washes to imitate mucociliary clearance. HRV-replication and mRNA expression was tested at indicated time-points. (**b**) Low-grade virus replication, apical shedding, and (**c**) the level of *IFNL1* mRNA during prolonged culture of HRV16 infected cells. (**d**) mRNA expression of *DNAI1*, *SPDEF*, *IFIT2,* and *MMP9* during persistent in vitro infection with HRV16. Data are shown as means and SD (n = 7). RM 2-way ANOVA (Sidak): *P < 0.05, **P < 0.01 in comparison to mock. (**e**) Graph summarizing bronchial epithelium responses in case of acute HRV infection and during virus persistence.

To see if such a remodeling-promoting phenotype persisted longer in the HRV infected epithelium, we analyzed responses to the virus in a simplified model of HRV persistence. The mucociliary differentiated epithelium was HRV-infected and next cultured for over two weeks with frequent removal of apical secretions and periodic surface washes (Fig. 4a). Prolonged culture resulted in a significant decrease in HRV16 replication and apical shedding (Fig. 4b; ~ 600-fold) with a concomitant decline of IFN-response (Fig. 4c). Nevertheless, we also observed continuous low-level virus replication (for at least 16 days) with only weak activation of the viral response and minor damage to the epithelium. Extended culture of HRV-infected epithelium was accompanied by almost complete normalization of mRNAs deregulated during the acute infection phase, including *FOXJ1* and *DNAI1*, which suggests a quick restoring of ciliogenesis (Fig. 4d; Supplementary Fig. S8a,b). Upregulated goblet cell markers also quickly returned to normal levels (e.g., *SPDEF* in Fig. 4d). Nevertheless, the expression of some antiviral (e.g., *IFIT2*, *OASL*) and structural genes (e.g., *MMP9*) remained moderately increased (~ twofold) during the ‘virus persistent’ phase indicating a putative mechanism preventing reinfection (Fig. 4d; Supplementary Fig. S8c). Additionally, in our model, both HRV replication and active IFN-responses were not prolonged in the epithelium exposed to inflammatory cytokines (Supplementary Fig. S9), which stays in line with clinical studies showing no difference in the duration of HRV shedding in asthma patients and control donors^27, 28^. Altogether, these data indicate that HRV-induced MCM and pro-fibrotic phenotype of the airway epithelium might be transient, yet recurrent or prolonged infections could have a significant impact on airway function (as summarized in Fig. 4e).

### Antiviral response of bronchial epithelium is similar in asthma patients and control subjects

Our results indicate that inflammatory conditions related to asthma influence the antiviral defense of the airway epithelium. There are, however, conflicting data in the literature regarding whether airway epithelial cells derived from asthma subjects have intrinsic defects in the antiviral response^17, 29–32^. To validate if such a discrepancy may result from phenotypic heterogeneity of asthma we compared the in vitro responses of bronchial epithelial cells derived from control subjects and asthma patients with different lower airway inflammation (clinical characteristics shown in Supplementary Table S1).

First, we compared baseline (i.e., day 34, w/o cytokines, not-infected) mRNA expression in ALI-grown bronchial epithelium from asthma patients and control donors (Supplementary Fig. S10a). Only minor differences in mRNA profile, i.e., a tendency toward a lower expression of ciliogenesis markers in asthma (Supplementary Fig. S10b,c), and a marked trend toward upregulation of mucous cell markers in epithelial cells derived from patients with eosinophilic asthma (Supplementary Fig. S10d,e), was observed. Nevertheless, the baseline expression of antiviral genes was the same in control subjects and asthma patients, irrespectively of the inflammatory phenotype of the disease (Supplementary Fig. S10b,d).

**Figure 5:**
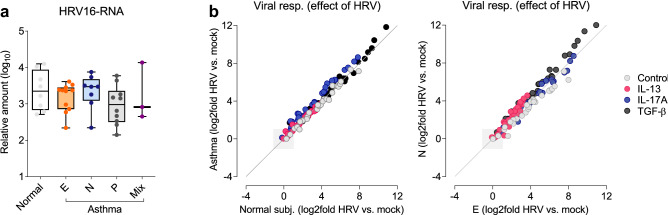
Airway epithelial responses to HRV infection are not impaired in asthma. (**a**) Similar replication of HRV16 in ALI-grown mucociliary epithelium from asthma patients with eosinophilic (E), neutrophilic (N), pauci-granulocytic (P) or mixed inflammatory (Mix) phenotypes. Data are presented as medians and range (n = 40; no difference with Kruskal–Wallis test). (**b**) HRV-related changes (log2fold data shown) in the expression of viral response genes were similar in cells from asthma patients and normal subjects (left panel), and in comparison of asthma patients with E and N inflammation in the lower airways (right panel).

Next, we analyzed the sensitivity to HRV in different subject groups. Interestingly, HRV16 replication and antiviral responses were similar in asthma patients and controls (Fig. 5a). Additionally, we did not observe any difference between the various asthma inflammatory phenotypes (Fig. 5b; Supplementary Fig. S11). Similarly, changes in the structural and remodeling genes (either due to cytokine stimulation or HRV16) were comparable in control subjects and asthma patients irrespectively of lower airway inflammation (data not shown). Therefore, our data suggest that changes in the epithelial response to HRV infection occur probably only in the presence of inflammation in vivo, and are not related to a putative genetic or epigenetic asthma signature maintained ex vivo.

## Discussion

Since the groundbreaking studies pointing to the diversity of airway inflammation in asthma^2, 4^, recent research has sought to understand how the immune mechanisms of the disease translate into the clinic. One crucial aspect is determining how T2 and non-T2 airway inflammatory endotypes modify the response to environmental triggers and whether they contribute differentially to the progression of structural changes in the airways. In this study, using in vitro culture of differentiated HBECs from asthma patients and control donors, we recreated different types of epithelial remodeling associated with key cytokines of asthmatic inflammation. Next, we used this model to analyze the expression of antiviral genes and susceptibility to HRV infection. Interestingly, MCM induced by IL-13, a canonical T2 cytokine, was associated with significant induction of viral response genes, including antiviral effectors related to type I and III IFN signaling. Since exposure to TGF-β increased the sensitivity to HRV, our data suggest that the outcome of viral infection depends both on the type of airway inflammation and the extent of epithelial damage and repair. Furthermore, we demonstrated that HRV infection per se resulted in a transient MCM, likely as an additional protective mechanism to self-limit the infection and prevent the virus from spreading.

The data presented here align with our previous findings showing decreased HRV replication in the airway epithelium after IL-13-induced MCM^17^. Another study reported on the decreased induction of type III IFNs in response to dsRNA in IL-13-stimulated airway epithelial cells^33^. Here, we expanded our knowledge about this issue, showing that IL-13-induced MCM is associated with an ‘antiviral state’ of bronchial epithelium, as evidenced by significant upregulation of viral response genes, particularly type III IFNs and ISGs. Together with a reduced number of ciliated cells, the two mechanisms likely contribute to the decreased vulnerability of MCM epithelium to HRV. Nevertheless, our study did not allow us to determine whether the antiviral state is already present in the mucous cells of the intact epithelium, or if it only develops during IL-13-induced MCM. However, data analysis from previous single-cell sequencing studies suggests an antiviral gene expression signature characterizing airway goblet cells^8, 34, 35^. For example, tracking cells in the lung cell atlas published by Vieira Braga et al.^8^ revealed enrichment of several ISGs (e.g., *ISG15*, *ISG20* and OAS-family) in the goblet cell subset (Supplementary Fig. S12). Similar patterns can be found both in data published by Deprez et al.^34^, who investigated single-cell gene expression in various airway locations, and in a recent survey of SARS-CoV-2 entry factors^35^ (Supplementary Fig. S13). Moreover, type I IFN gene expression signature was exceptionally high in the nasal epithelium^36^, especially in a subset of goblet cells^37^, indicating their putative conditioning to reduce susceptibility to viral infections. Interestingly, in our study, *ISG15*, *ISG20*, and OAS-like transcripts were also the top ISGs upregulated during IL-13-induced MCM. Altogether, the previously published and our results indicate that airway mucous cells are characterized by a gene expression profile suggesting a more robust antiviral defense, which can be further enhanced during IL-13-induced MCM.

Investigating how non-T2 inflammatory processes modify the antiviral responses of airway epithelial cells is much more challenging. In contrast to the well-defined T2 subtype associated with eosinophilic inflammation, various immune mechanisms were implicated in the pathobiology of non-T2 asthma^23^. In this study, we used IL-17A stimulation to substitute the non-T2 conditions associated with a neutrophilic variant of asthma^22^. Interestingly, exposure of bronchial epithelium to IL-17A resulted in an opposite effect compared to IL-13, with downregulation of most genes involved in the antiviral defense. IL-17A also led to a significant reduction of ciliogenesis in our model, which explains why HRV replication did not substantially increase in that setting compared to control conditions.

Based on the presented data, we might hypothesize that eosinophilic asthma, which develops on a T2-immune background, should not increase the risk of severe infections with respiratory tract viruses. This issue has not been extensively studied until the recent outbreak of COVID-19. Contrary to expected, the diagnosis of asthma was not associated with higher susceptibility to SARS-CoV-2 infection^38^, nor with a worse clinical outcome^39^. One explanation could be the lower epithelial expression of ACE2, a SARS-CoV-2 entry receptor, in asthma patients with T2-high airway inflammation^40, 41^. Since the innate defense of airway epithelium is very similar in response to various RNA viruses^41^, the ‘antiviral state’ linked with T2-inflammation shown in our study, may in general protect against severe outcomes during infections with respiratory viruses. The downside of this mechanism might be the concurrent hypersecretion of mucus, which could impair mucociliary clearance and thus increase the risk of airway obstruction. Further clinical studies are required to validate how T2 and non-T2 inflammation affect the frequency and severity of respiratory virus infections in asthma.

Nevertheless, our study documents an important mechanism that may counteract the protective effect of T2 immune conditions. It refers to the role of growth factors during repair and remodeling of bronchial epithelium. As it turned out, TGF-β facilitated the replication of HRV, further aggravating the innate immune response associated with virus infection. That observation is in line with earlier studies showing that exposure to TGF-β significantly promoted the replication of HRV both in primary airway epithelial cells^42^ and lung fibroblasts^43^. Furthermore, in influenza virus-infected mice, intrabronchial administration of growth factors worsened the course of respiratory tract illness^44^. The reason why TGF-β-exposed airway epithelial cells are more sensitive to viral infections is not well understood. Yet, it may be due to their phenotypic similarity to undifferentiated or regenerating epithelial cells. Some explanation has been provided by studies that tracked changes in gene expression during cell differentiation in the ALI system^45–47^. In that setting, primary HBECs cells showed the low expression of host defense genes, such as *IFITM1*, *MX1*, and *IFIT3*, which gradually increased during mucociliary differentiation^47^. Altogether, these and our data suggest that exposure of bronchial epithelium to growth factors, for example during epithelial repair^48^, may result in pronounced HRV replication and more robust innate response. Indeed, earlier studies showed that proliferating primary bronchial epithelial cells can be readily infected by HRV^17, 49^, respiratory syncytial virus^50^, or influenza virus^51^. Similarly, exposure of basal cells in the injured epithelium resulted in a much higher sensitivity to HRV^52^. In consequence, the areas of airway epithelial damage, which are a common finding in the lower airway of asthma patients, could serve as the entry site for HRV and enhance virus replication, likely promoting infection-related asthma exacerbation. Therefore, the potential protective effect of T2 immune conditions may no longer operate in patients with more advanced epithelial lesions, e.g., with severe asthma, or after exposure to inhaled noxious agents, such as environmental smoke.

Interestingly, the response to HRV infection was similar in cells derived from asthma patients and control individuals. There was also no difference in comparison of asthma patients with eosinophilic and neutrophilic airway inflammation. There are conflicting data in the literature concerning this issue. Earlier reports showed defective type I and III IFN production by HRV infected bronchial epithelial cells derived from asthma patients^29, 30^, suggesting a global defect in IFN pathway. Conversely, more recent studies did not confirm that observation either in primary cell lines^31, 53^ or in ALI-cultures^17, 32^. Thus, our results stay in line with earlier data obtained from polarized nasal epithelium by Bai et al.^19^, and indicate that altered susceptibility of the airway epithelium to the virus is not related to a putative genetic or epigenetic ‘asthmatic’ signature but becomes apparent only in the presence of overt inflammation or epithelial cell structural changes.

In the final part of the study, we examined whether HRV by itself could induce remodeling of the bronchial epithelium. For this purpose, we assessed the expression of genes related to the epithelial structure. We also checked how long-term culture, which to some extent reflects recurrent or persistent infections in humans, would affect the antiviral response and epithelial function. Interestingly, HRV infection markedly increased the expression of *MUC5AC* and other MCM markers. Additionally, we demonstrated an upregulation of genes involved in airway remodeling, notably growth factors FGF2 and EGF. Our results are consistent with previously published data showing an increased number of goblet cells and MUC5AC expression in HRV infected polarized airway epithelium^19, 54^, and explains the associated mucous hypersecretion^54, 55^. Similarly, increased expression of *FGF2* in response to HRV has been described both in primary airway epithelial cell^56, 57^ and in polarized airway epithelium^58, 59^ suggesting that infected bronchial epithelial cells, by production of growth factors acting on fibroblasts, may also contribute to stroma cell proliferation. Taken together, these data indicate that HRV infection promotes MCM of the bronchial epithelium, but at the same time it may contribute to the release of growth factors that aid in the regeneration of the epithelium, yet inducing a potentially pro-fibrotic phenotype of the tissue.

Importantly, our data show that HRV infection of bronchial epithelium is efficiently self-limited in vitro, irrespectively of inflammatory cytokine stimulation, which suggests an exceptional self-sustaining property of the tissue. It stays in line with a recent study by Essaidi-Laziosi et al.^21^, who demonstrated transient innate activation in HRV infected nasal epithelial cells, followed by a virus persistence phase with contained cell responses and associated tissue recovery. Nevertheless, we showed low-grade HRV replication in the prolonged culture, accompanied by a weak innate immune response, suggesting that persistent HRV infections can develop under certain clinical conditions, e.g., in case of immature or deficient immunity. Indeed, extended HRV shedding was reported in infants^28, 60, 61^, in elderly^62^, and immunocompromised patients^28, 63, 64^. Although the impact of medication was beyond the scope of our study, it has been shown that glucocorticoids enhance the replication of HRV in vitro and delay virus clearance^65, 66^. Similar mechanisms may occur in patients with severe asthma receiving high doses of inhaled or systemic corticosteroids^65, 66^. Interestingly, HRV was frequently detected in the airways of asymptomatic subjects, particularly among young children^10, 11^. In such cases, virus positivity was accompanied by a gene expression profile indicating the antiviral response of epithelium^41, 67^. That evokes an intriguing hypothesis that prolonged periods of the ‘antiviral state’ in the airways due to HRV persistence or asymptomatic infections may be actually an evolutionary host–pathogen adaptation mechanism to prevent deleterious infections with more serious viral pathogens^68^.

In conclusion, our data suggest that the bronchial epithelial cell response to HRV infection depends on the type of lower airway inflammation and the extent of epithelial damage. The MCM associated with T2-inflammation produces an antiviral state and therefore has a protective effect by limiting virus replication and the magnitude of innate response. In addition, HRV infection itself can stimulate MCM and induce a transient pro-fibrotic phenotype of the tissue, which in the case of repeated or persistent infections poses a potential risk factor of airway remodeling.

## Methods

Human bronchial epithelial cells (HBECs) were isolated from bronchial biopsies obtained during bronchoscopy (Supplementary Fig. S1) in asthma patients (n = 32, primarily severe), and in control, non-asthma subjects (n = 8). Clinical and demographic characteristics are presented in Supplementary Table S1. Cells were differentiated 26 days in an air–liquid interface transwell system (Corning Inc., Corning, NY), and next incubated an additional 8 days with IL-13, IL-17A, or TGF-β_1_ (all from R&D Systems, Minneapolis, MN) in a model of chronic cytokine stimulation (Fig. 1a). Control and cytokine-exposed epithelia were infected with HRV16 at a fixed quantity of 10^6^ plaque-forming units (PFU) per insert. Virus replication (5’UTR specific probe; TIB-Molbiol, Berlin, Germany), and mRNA expression (66 targets; i.e., antiviral response genes and structural/remodeling genes, Supplementary Table S2) were analyzed 48 h post-infection using the real-time PCR system (Quant Studio 12K Flex Real-Time PCR System, Applied Biosystems). Epithelial responses were initially assessed in the whole study group (all data combined), and next between cell lines derived from asthma patients and non-asthmatic donors. Some analyses (e.g., initial mRNA screening) were performed in fewer cell lines (n = 19). We also performed additional experiments to study HRV responses during cytokine-induced remodeling (n = 4), and virus persistence (n = 7). The investigation was carried out in accordance with the Declaration of Helsinki. The study protocol was approved by Bioethics Committee of the Jagiellonian University and informed written consent was obtained from each participant. A full description of the methods is presented in the Online Repository.

## Supplementary Information

Supplementary Information.
