# Supplementary material for: Remodeling of bronchial epithelium caused by asthmatic inflammation affects its response to rhinovirus infection
Source: Sci Rep. 2021 Jun 17;11:12821. doi: 10.1038/s41598-021-92252-6 (PMC8211645; doi:10.1038/s41598-021-92252-6)
Supplement: Supplementary file 1 — Supplementary Information. [file 41598_2021_92252_MOESM1_ESM.pdf]

Supplementary Information

Remodeling of bronchial epithelium caused by asthmatic inflammation affects its response to rhinovirus infection

Bogdan Jakiela*, Ana Rebane, Jerzy Soja, Stanislawa Bazan-Socha, Anet Laanesoo, Hanna Plutecka, Marcin Surmiak, Marek Sanak, Krzysztof Sladek, and Grazyna Bochenek

* Corresponding author
Bogdan Jakiela, MD, PhD
Department of Internal Medicine, Jagiellonian University Medical College
Skawinska 8, 31-066 Kraków, Poland
e-mail: b.jakiela@uj.edu.pl

Contents

1. Supplementary Data: Materials and methods .... Page 2
2. Supplementary Tables .... Page 7
3. Supplementary Figures .... Page 10
4. References .... Page 23

1. Materials and methods

Characteristics of subjects studied

In this study, we used cryopreserved primary human bronchial epithelial cell (HBECs) lines (cryo-bank repository) that were isolated from brush biopsies and/or microbiopsies of bronchial mucosa obtained during bronchoscopy. The investigation was carried out in accordance with the Declaration of Helsinki. Study subjects were recruited at the Department of Internal Medicine of the Jagiellonian University Medical College, Krakow, Poland. Cells were retrieved during our earlier projects with patient sampling procedure and study protocols approved by Bioethics Committee of the Jagiellonian University (KBET/68/B/2008 and KBET/284/B/2014) and informed written consent obtained from each participant. Overall, we performed experiments in HBECs isolated from 32 asthma patients and 8 normal subjects. The control group consisted of patients referred for bronchoscopy due to the diagnosis of cough. In these subjects, asthma, respiratory tract infections, or other diseases of the respiratory tract were ruled out during the procedure and further clinical investigation. All subjects enrolled were current non-smokers. Asthma diagnosis and severity was assessed according to the GINA guideline.[1] Additionally, lung function at baseline and after bronchodilation (MasterScreen, Jaeger, Wurzburg, Germany), allergy status (skin prick testing with common allergens; Allergopharma, Reinbeck, Germany), blood eosinophil count (Sysmex Co., Kobe, Japan), serum IgE (BNII, Siemens Healthineers, Munich, Germany), and serum Periostin (Biovision Inc., Milpitas, CA) was assessed in all participants. Detailed characteristics of the subjects are presented in Supplementary Table S1. Fisher's exact test (categorical data) and Kruskall-Wallis model (Dunn's post hoc test) were used to compare group characteristics. The groups did not differ in demographic parameters, but compared to normal donors, asthma patients showed airway obturation, increased blood eosinophil count, and serum IgE concentration.

The type of lower airway inflammation was assessed by examination of bronchoalveolar lavage fluid (BALF) cell differential count (Supplementary Fig. S1). In brief, during the bronchoscopy procedure, the fiberscope was wedged in the segmental bronchus of the right middle lobe and 0.9% saline (200 mL in portions) was instilled to recover BALF. Cell differential was determined by counting ≥1000 cells in MGG stained cytospin slides (Thermo Fisher Scientific, Walthman, MA). The numbers of inflammatory cells in BALF did not differ between asthma patients and control subjects (except for a trend [P=0.1] toward a higher percentage of eosinophils in asthma). Based on the percentage of BALF cells, we identified lower airway inflammatory phenotypes of the disease using the upper limits reported for normal subjects:[2–4] eosinophilic (E; >2% eosinophils, <3% neutrophils), neutrophilic (N; <2% eosinophils, >3% neutrophils), mixed inflammatory (Mix; >2% eosinophils, >3% neutrophils), and paucigranulocytic (inflammatory cells within normal range). The distribution of inflammatory phenotypes corresponded to published data with approximately 45% patients showing airway eosinophilia, and 25% N-phenotype (Supplementary Fig. S1bc).[5–7] Each HBEC line was therefore fully characterized in terms of clinical status and type of lower airway inflammation of the patient.

For some initial analyses, e.g., screening of mRNA expression in different culture conditions, we used a smaller number of cell lines (n=19, including 13 asthmatics and 6 controls). To enable

comparison of the main inflammatory phenotypes of the disease, only asthma patients with E (n=7) and N (n=6) airway inflammation were included.

Isolation of bronchial epithelial cells and cell cultures

HBECs were isolated from bronchial biopsies (e.g., brushing) by enzymatic digestion (pronase and DNAse; all cell culture reagents from Sigma-Aldrich [Merck], St. Louis, MO), cultured in type IV collagen-coated flasks in supplemented bronchial epithelial growth medium (BEGM, all media from Lonza, Basel, Switzerland) and next cryopreserved (Supplementary Fig. S1a). In all experiments we used polarized bronchial epithelium differentiated *in vitro* using a transwell system (Costar, Corning Inc., Corning, NY). In brief, primary HBEC (cryopreserved cells expanded as passage 1 or 2) were seeded onto transwell membranes in 12-well plates at the density 0.15×10^6 cells/cm^2, grown initially submerged (48 h) in supplemented BEGM, and next switched to air-liquid interface (ALI) and ALI-medium, i.e. 1:1 mixture of Dulbecco's modified Eagle's and BEGM with supplements and all-*trans*-retinoic acid (75 nM). After 2-3 weeks of ALI culture, cells developed mucociliary features which was confirmed by immunofluorescent staining of ciliated and goblet cells, as previously described.[8] To ensure similar conditions, day-26 of the ALI culture was used as a starting point for all further experiments. Excess of surface secretions and mucus were removed with gentle suction during media changes.

Cytokine-induced changes in the bronchial epithelium were analyzed using a model of chronic cytokine treatment (Supplementary Fig. S2a). Day-26 ALI-cultures were grown 8 days in the presence of IL-13, IL-17, or TGF-β (all from R&D Systems, Minneapolis, MN) added to the baso-lateral medium (10 ng/mL or lower in the titration experiments [Supplementary Fig. S3de]) with media changes every other day. During infection experiments, human rhinovirus-16 (HRV16, or mock solution) was added to the apical side 2 d before the ending of cytokine stimulation and harvesting of cells. On day 34 (ALI), cells were mixed with TRI-reagent (Sigma-Aldrich) for further RNA extraction. Selected membranes were fixed with paraformaldehyde (PFA) for immunofluorescent (IF) staining. The baso-lateral medium was aliquoted and stored at -80°C for further analysis of cytokine concentration. Any modifications of cell cultures are described in the manuscript text and figure legends.

HRV infection of ALI-differentiated bronchial epithelial cells

HRV16 was replicated in HeLa cells (European Collection of Cell Cultures, Salisbury, UK), concentrated by centrifugation, and titrated with a standard plaque assay. In brief, serial $\log_{10}$ dilutions (in PBS containing Ca and Mg) were incubated 30 min with HeLa cells in 6-well plates (seeded at density 0.9×10^6 cells/well), next covered with 0.8% noble agar (reagents from Sigma-Aldrich) with modified minimal essential medium and incubated 4 days at 34°C. Plaques were visualized after PFA fixation and crystal violet staining. Virus stocks were adjusted to the required number of plaque-forming units (PFU) per volume with PBS-Ca/Mg containing 0.25% BSA and stored at -80°C. The identity of virus strain propagated in our lab (originally from Wai-Ming Lee, Wisconsin University) was confirmed by sequencing and phylogenetic analysis.

Before infection, the surface of the epithelium was washed with PBS-Ca/Mg (5-times, 0.7 mL each) to remove the mucus, and the cell culture medium was switched to ALI-medium with 100-fold reduced concentration of hydrocortisone (HC). In each infection experiment, we used a fixed amount of HRV16, i.e., 10^6 PFU in 0.1 mL of PBS/BSA (per 1 insert of 12-well plate), which was added to the apical epithelial surface and next incubated for 30 min at room temperature and 1.5 h at 34°C in the cell culture incubator (Fig. 2a). After 2 h attachment, the surface was extensively washed with PBS-Ca/Mg (8-times, 0.7 mL each) to remove as many unbound HRV16 particles as possible. The residual amount of virus usually did not exceed 10^4 genome copies per insert; it was thus 1,000 to 10,000-lower than the final titers (Fig. 2b) and should not influence results. Uninfected wells (mock) in a parallel plate were exposed to 0.1 mL of diluent alone (PBS/BSA); otherwise, they were treated the same way as infected cells, including all washing steps. The newly replicated HRV was assessed 48 h post-infection in the surface fluid and cell lysates. Apical secretions, which also contained cell debris, were mixed with 0.5 mL of PBS-Ca/Mg by gentle pipetting; a fixed volume (50 µL) was then mixed with TRI-reagent and immediately frozen at -80°C. Similarly, cells in the insert were mixed with TRI-reagent and stored for further RNA isolation. Total RNA was extracted using silica column kits (A&A Biotechnology, Gdynia, Poland) and reverse transcribed (High Capacity cDNA Reverse Transcription Kit; Applied Biosystems, Foster City, CA).

Complementary experiments were performed using a smaller number of cell lines. For example, the analysis on how structural changes modulate sensitivity to HRV16 (shown in Supplementary Fig. S3a-c) was investigated using 4 cell lines (2 from control subjects, 2 from asthma patients). Day 26 ALI-cultures were treated 8-days with cytokines (control conditions, IL-13, IL-17A, or TGF-β_1, 10 pg/mL each) and infected with HRV16 at different timepoints, i.e., during incubation with cytokines (days 0, 2, 6) and after cytokine withdrawal (days 8 and 16). The procedure of infection was, however, the same as in the main experiments. Model of prolonged HRV16 infection (shown in Fig. 4a and Supplementary Fig. S8 and S9) was tested in 7 cell lines (3 from control subjects, 4 from asthma patients). Cells were mucociliary differentiated in the ALI system for 32 days (two 12-well plates). Epithelia were next apically infected with HRV16 (10^6 PFU/well) using the same procedure described above. First samples were collected on day 2 after infection to assess the initial replication and epithelial response, reflecting the same conditions as in the main experiment. Cells were next cultured for an additional two weeks with ALI-medium (10-fold reduced HC) changed every other day. Additionally, to imitate mucociliary clearance (removal of cell debris and released virus), the surface was gently washed with PBS-Ca/Mg (3-fold, 0.7 mL each) every four days. Apical secretions and cell lysates were collected from HRV16 or mock-treated cells at days 8 and 16 since infection to determine the rate of HRV replication and mRNA profile. The exact numbers of replicates are presented in figure legends for clarity.

Quantification of HRV16

Amount of HRV16 was quantified separately in apical secretions (labeled 'HRV-Titer' in graphs) and cell lysates (labeled 'HRV16-RNA' in graphs) by Fast Real-time PCR using 5'UTR specific primers (5'-CCTCCggCCCCTgAATgTg, 5'-gAAACACggACACCCAAAgTA) and TaqMan 6-FAM probe (5'-

TggTCgTAATgAgCAATTgCgg; oligonucleotide synthesis by TIB-Molbiol, Berlin, Germany). Apical fluid qPCR data were adjusted to the standard curve (generated with a purified amplicon) and accounted for sequential dilutions; thus presented as the number of HRV16 genome copies in 5 µL of collected apical fluid (i.e., corresponding to 1% of all viral particles or viral RNA released apically in a given epithelial culture). The detection limit was ~10 copies of HRV genome per reaction (corresponding to cycle 36.8 in real-time PCR). When comparing the infectability of the inoculum and PCR results, 1 PFU corresponded to approximately 400-500 copies of the viral genome (i.e., virus particles).

Immunofluorescent staining of ciliated and goblet cell markers

Pieces of insert membranes with PFA-fixed cells were permeabilized with 0.3% Triton-X100 (all reagents from Sigma-Aldrich, if not otherwise specified), blocked with goat IgG and FCS and incubated with primary mouse monoclonal antibodies anti-MUC5AC (#M5293) or anti-β-Tubulin-IV (#T7941), followed by secondary staining with goat anti-mouse IgG Fab labeled with Alexa Fluor 488 or Alexa Fluor 568 (both from Invitrogen, Carlsbad, CA) and Hoechst 33342 to counterstain cell nuclei. Mounted slides were imaged with the epifluorescent microscope (Axio Scope, Carl Zeiss, Jena, Germany). The same areas were photographed (at 400x magnification) with different sets of filters and digitally overlaid using image processing software (Corel Photo-Paint X8, Corel Corporation, Ottawa, Canada). The number of goblet or ciliated cells (i.e., marker positive) per surface area was averaged after counting cells in 10 images (from different insert membrane areas) per sample. Image collection and cell counting were performed in a standardized manner by a blinded investigator. Our initial screen showed a striking correlation between the number of ciliated and goblet cells (counted in IF slides) with their respective gene expression markers *DNAI1* and *MUC5AC* (Supplementary Fig. S2c).

Analysis of mRNA expression with real-time PCR

For gene expression analysis total cellular RNA was isolated using Total RNA Zol-Out Kit (A&A Biotechnology), purified with Clean-Up RNA Concentrator Kit (including DNAse treatment step, A&A Biotechnology), and reverse-transcribed with random hexamers (High Capacity cDNA Reverse Transcription Kit, Applied Biosystems). Relative mRNA expression was analyzed with TaqMan low-density arrays (TLDA, Applied Biosystems) or single TaqMan assays using QuantStudio 12K Flex Real-Time PCR System (Applied Biosystems). A list of all mRNA gene targets and corresponding TaqMan assays, together with the assignment of major gene expression signatures (e.g., ciliated cells) is presented in Supplementary Table S2. Raw results were processed using ExpressionSuit Software v1.1 (Thermo Fisher Scientific, Massachusetts, MA) and normalized to *GAPDH*. Data are thus presented as a relative expression in comparison to *GAPDH* (x1000, $\log_{10}$); to simplify we use “Relative expression ($\log_{10}$)” labels in graphs. Relative quantities of individual transcripts in comparison to different conditions were calculated using a $2^{-\Delta\Delta CT}$ method. At least 2-fold change in mRNA expression (i.e., log2fold <-1 or >1) was considered biologically significant. In V-plots, log2fold values were plotted against $-\log_{10}P$-values estimated with a 2-sided paired t-test. Results were controlled for multiple comparisons using the

Benjamini-Hochberg correction at the false discovery rate threshold (FDRt) q=0.05. Initially, we analyzed a larger number of mRNA transcripts (66 targets) in a smaller set of samples (n=19, including 13 asthmatics). These data are shown in summary diagrams, such as V-plots, Venn-plots, and heat maps. Expression of selected genes (34 targets) was validated in the whole study group (n=40), which also included the comparison of asthma subtypes. For convenience, mRNA expression was analyzed in two sets of assays: (1) genes linked with antiviral responses, and (2) genes related to epithelial structure and remodeling, as assigned in Table S2. For clarity, in the first part of the study we present data on the expression of antiviral genes (shown in Figs 1 and 2), whereas in the further sections we depict the changes in the expression of structural and remodeling-related genes (summarized in Fig. 3).

Concentration of cytokines in the cell culture supernatant

Levels of IL-29 and IFN-β were assessed in the baso-lateral medium using IL-29 (IFN lambda 1) Uncoated Elisa Kit (#88-7296-88, lowest standard 8 pg/mL; Invitrogen) and Human IFN-beta Duo-Set ELISA (#DY814-05, lowest standard 7.8 pg/mL; R&D Systems) according to manufacturer protocols.

Statistics

Statistical analysis was performed with Statistica 13.1 (TIBCO Software Inc., Palo Alto, CA) and GraphPad Prism 8.4 (GraphPad Software, Inc., La Jolla, CA). Individual data points and means with standard deviation (SD) ranges are shown in graphs, except for time-course experiments (for clarity only means and SD or standard error of the mean [SEM], e.g., Fig. 4 in the main body of the manuscript), and clinical group comparisons (median and range, e.g., Fig. 5). 2-sided paired t-test or RM (repeated measures) 1-way ANOVA was used in most of comparisons, unless otherwise specified. For multivariate analyses, data were normalized by log-transformation and mean zeroing. Hierarchical clustering of expression data (Ward's method based on Pearson's coefficients in most instances) and principal component analysis were performed with the Statistica package. Heat maps of cross-correlations are based on Pearson's coefficients (R_P), with only the most significant trends (P<0.01) highlighted with asterisks. Data trends in plots showing correlations (e.g., between gene expression and HRV replication shown in Supplementary Fig. S6b) are highlighted with 4-knot spline lines. The Benjamini-Hochberg procedure was used to correct for multiple testing errors in mRNA expression analyses with the false discovery rate threshold (FDRt) set at q=0.05. Significant differences are marked in graphs with symbols: ∗ P-value <0.05 but ≥0.01, ∗∗P<0.01, unless otherwise stated. We paid attention to present information on the number of replicates, data presentation format, and statistical tests in each figure legend.

2. Supplementary Tables

Table S1. Clinical characteristics of the subjects in the validation part of the study (n=40).

	**Control** n=8	**Asthma** n=32	*P*-value
Age (years)	34.5 [29.5-46]	47.5 [37-57.5]	0.083
Females (n, %)	2 (25%)	17 (53.1%)	0.24
Asthma onset (at age)	n.a.	30 [21-43]	n.a.
Asthma duration (years)	n.a.	15.5 [5.3-25]	n.a.
Early onset (n, %)	n.a.	6 (18.8%)	n.a.
Severe asthma (n, %)	n.a.	21 (65.6%)	n.a.
iGCS (µg/d), range #	n.a.	1000 [250-4000]	n.a.
sGCS (n, %)	none	10 (31.3%)	n.a.
sGCS (mg/d), range ∗	n.a.	0 [0-32]	n.a.
LABA (n, %)	none	29 (90.6%)	n.a.
LTRA (n, %)	none	6 (18.8%)	n.a.
FEV_1%FVC	80.3 [76.6-83.0]	68.2 [56.9-76.8]	**0.0002**
FEV_1%FVC after bronchodilator	82.7 [79.7-84.9]	73.7 [59.7-79.9]	**0.007**
FEV_1% predicted	107 [104-116]	72 [59-94.8]	**0.0005**
FEV_1 reversibility ≥12% (n,%) §	none	11 (35.5%)‡	n.a.
Positive skin prick tests (n, %)	1 (12.5%)	17 (53.1%)	0.053
Serum total IgE (IU/mL)	33.8 [18.9-52.2]	101.5 [67.9-210.3]	**0.005**
Blood eosinophils (cells/µL)	130 [52-216]	309 [128-634]	**0.039**
Blood Periostin (ng/mL)	49 [32.4-56]	50.4 [30.5-58.2] †	0.82
Lower airway inflammation			
BALF Neutrophils (%)	1.4 [0.3-2.1]	1.7 [0.6-3.5]	0.27
BALF Eosinophils (%)	0.2 [0-0.3]	1.0 [0-10.4]	0.1
BALF Lymphocytes (%)	6.8 [3.5-13.1]	5.6 [4.1-10.9]	0.91
Inflammatory phenotype: E/N/P/Mix	n.a.	11/8/10/3	n.a.

Table S1 footnotes:
Abbreviations: BALF – broncho-alveolar lavage fluid, FEV_1 – forced expiratory volume in 1 second, FVC – forced vital capacity, iGCS – inhaled glucocorticoids, sGCS – systemic glucocorticoids, LABA – long-acting β_2-agonist, LTRA - leukotriene receptor antagonist, n.a. – non applicable. Lower airway inflammatory phenotypes: E – eosinophilic, N – neutrophilic, P – pauci-granulocytic, Mix – mixed inflammatory.
References: # – all asthma patients were treated with iGCS (the dose adjusted for fluticasone), ∗ – dose adjusted for methylprednisolone, § - after inhalation of short-acting β_2-agonist, ‡ – FEV1-reversibility data not available for one subject, † – concentration of blood Periostin available only in 21 (66%) asthma patients.
Statistics: Data are presented as medians [25-75 quartiles] unless otherwise expressed. Contingency table statistics (Fisher's exact test) and Mann-Whitney test statistics (if applicable) are shown in a separate column. Significant differences (P<0.05) are additionally highlighted with a bold font.

Table S2. List of TaqMan assays used for analysis of mRNA expression.

	Assay ID*	Gene Symbol	Gene Name	mRNA signature
			Viral response genes	
1	Hs00171042_m1	*CXCL10*	C-X-C motif chemokine ligand 10	Chemokine
2	Hs00174103_m1	*CXCL8*	C-X-C motif chemokine ligand 8	Chemokine
3	Hs01061436_m1	*DDX58*	DEXD/H-box helicase 58 (RIG-1)	dsRNAs
4	Hs01597843_m1	*DHX58*	DEXH-box helicase 58 (LGP2)	dsRNAs
5	Hs00169345_m1	*EIF2AK2*	eukaryotic translation initiation factor 2 alpha kinase 2	ISGs
6	Hs00977005_m1	*GBP1*	guanylate binding protein 1	ISGs
7	Hs00164932_m1	*ICAM1*	intercellular adhesion molecule 1	ICAM-1
8	Hs00223420_m1	*IFIH1*	interferon induced with helicase C domain 1 (MDA5)	dsRNAs
9	Hs03027069_s1	*IFIT1*	interferon induced protein with tetratricopeptide repeats 1	ISGs
10	Hs01922738_s1	*IFIT2*	interferon induced protein with tetratricopeptide repeats 2	ISGs
11	Hs00705137_s1	*IFITM1*	interferon induced transmembrane protein 1	ISGs
12	Hs01066116_m1	*IFNAR1*	interferon alpha and beta receptor subunit 1	IFN rec.
13	Hs01077958_s1	*IFNB1*	interferon beta 1	IFNs
14	Hs00601677_g1	*IFNL1*	interferon lambda 1	IFNs
15	Hs00820125_g1	*IFNL2*	interferon lambda 2	IFNs
16	Hs00417120_m1	*IFNLR1*	interferon lambda receptor 1	IFN rec.
17	Hs00971965_m1	*IRF1*	interferon regulatory factor 1	IRFs
18	Hs01014809_g1	*IRF7*	interferon regulatory factor 7	IRFs
19	Hs01921425_s1	*ISG15*	ISG15 ubiquitin-like modifier	ISGs
20	Hs00158122_m1	*ISG20*	interferon stimulated exonuclease gene 20	ISGs
21	Hs00895608_m1	*MX1*	MX dynamin like GTPase 1	ISGs
22	Hs01550814_m1	*MX2*	MX dynamin like GTPase 2	ISGs
23	Hs00942643_m1	*OAS2*	2'-5'-oligoadenylate synthetase 2	ISGs
24	Hs00196324_m1	*OAS3*	2'-5'-oligoadenylate synthetase 3	ISGs
25	Hs00984387_m1	*OASL*	2'-5'-oligoadenylate synthetase like	ISGs
26	Hs00369813_m1	*RSAD2*	radical S-adenosyl methionine domain containing-2 (Viperin)	ISGs
27	Hs01551078_m1	*TLR3*	toll like receptor 3	TLRs
28	Hs01933259_s1	*TLR7*	toll like receptor 7	TLRs
			Epithelial structure and remodeling genes	
1	Hs00426835_g1	*ACTA2*	actin, alpha 2, smooth muscle, aorta	EMT signat.
2	Hs00269932_m1	*ASCL1*	achaete-scute family bHLH transcription factor 1	PNECs
3	Hs00171082_m1	*CCL24*	C-C motif chemokine ligand 24	Other
4	Hs00171146_m1	*CCL26*	C-C motif chemokine ligand 26	Other
5	Hs00219797_m1	*CCL28*	C-C motif chemokine ligand 28	Other
6	Hs01023895_m1	*CDH1*	cadherin 1	EMT signat.
7	Hs00983056_m1	*CDH2*	cadherin 2	EMT signat.
8	Hs00357011_m1	*CFTR*	cystic fibrosis transmembrane conductance regulator	Ionocyte
9	Hs00976287_m1	*CLCA1*	chloride channel accessory 1	Other
10	Hs00221623_m1	*CLDN1*	claudin 1	TJC signat.
11	Hs00164004_m1	*COL1A1*	collagen type I alpha 1	EMT signat.
12	Hs01548179_m1	*CRB3*	crumbs 3, cell polarity complex component	TJC signat.
13	Hs00170014_m1	*CTGF*	connective tissue growth factor	Other
14	Hs00201755_m1	*DNAI1*	dynein axonemal intermediate chain 1	Ciliated cell
15	Hs01099990_m1	*EGF*	epidermal growth factor	EMT signat.
16	Hs00266645_m1	*FGF2*	fibroblast growth factor 2	EMT signat.
17	Hs01549976_m1	*FN1*	fibronectin 1	EMT signat.

18	Hs00270130_m1	*FOXA3*	forkhead box A3		Goblet cell
19	Hs00201827_m1	*FOXI1*	forkhead box I1		Ionocyte
20	Hs00230964_m1	*FOXJ1*	forkhead box J1		Ciliated
21	Hs01073586_m1	*FOXM1*	forkhead box M1		Other
22	Hs04931857_m1	*IL33*	interleukin 33		Other
23	Hs00957562_m1	*MMP9*	matrix metallopeptidase 9		EMT signat.
24	Hs01365616_m1	*MUC5AC*	mucin 5ac, oligomeric mucus/gel-forming		Goblet cell
25	Hs00861595_m1	*MUC5B*	mucin 5B, oligomeric mucus/gel-forming		Other
26	Hs00170162_m1	*OCLN*	occludin		TJC signat.
27	Hs01566750_m1	*POSTN*	periostin		Other
28	Hs00940851_m1	*SLC16A7*	solute carrier family 16 member 7		SLC16A7+
29	Hs00195591_m1	*SNAI1*	snail family transcriptional repressor 1		EMT signat.
30	Hs00161904_m1	*SNAI2*	snail family transcriptional repressor 2		EMT signat.
31	Hs00171942_m1	*SPDEF*	SAM pointed domain containing ETS transcription factor		Goblet cell
32	Hs00998133_m1	*TGFB1*	transforming growth factor beta 1		EMT signat.
33	Hs00978340_m1	*TP63*	tumor protein p63		Basal cell
34	Hs00175822_m1	*TRPM5*	transient rec. potential cation channel subf. M member 5		Tuft cell
35	Hs00263639_m1	*TSLP*	Thymic stromal lymphopoietin		Other
36	Hs01675818_s1	*TWIST1*	twist family bHLH transcription factor 1		EMT signat.
37	Hs00958111_m1	*VIM*	vimentin		EMT signat.
38	Hs00207691_m1	*ZEB2*	zinc finger E-box binding homeobox 2		EMT signat.

Table S2 footnote: * - TaqMan gene expression assays were purchased from Applied Biosystems (Foster City, CA, USA). Hs_99999905_m1 (*GAPDH*) was used as a reference gene. Gene symbols according to EnterezGene, NCBI (http://www.ncbi.nlm.nih.gov/gene/), accessed Oct 13, 2020.

Abbreviations: dsRNAs – double-stranded RNA sensors, EMT – epithelial-mesenchymal transition, IFN – interferon, IRFs – interferon regulatory factors, ISGs – interferon-stimulated genes, PNECs – pulmonary neuroendocrine cells, TJC – tight junction complex, TLRs – toll-like receptors.

The colors depicted here have also been used in the figures to denote the corresponding mRNA signatures.

3. Supplementary Figures

Figure S1

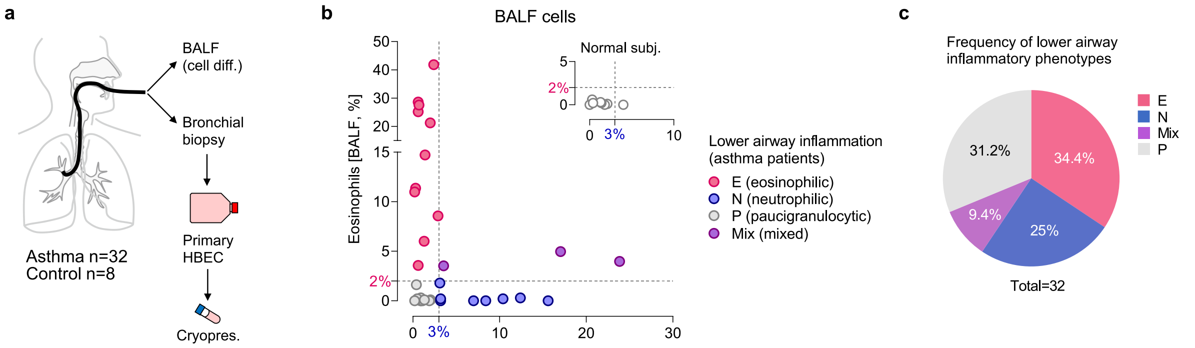

Figure S1 legend. Lower airway inflammatory phenotypes in enrolled asthma patients. (**a**) Bronchoalveolar lavage fluid (BALF, retrieved from the right middle lobe) and bronchial mucosa biopsies were sampled during bronchoscopy performed in asthma patients (n=32) and control subjects (n=8). After enzymatic digestion and dispersion of the tissue sample, primary bronchial epithelial cells (HBEC) were grown until sub-confluent in collagen-coated T-flasks and next cryopreserved for further experiments in air-liquid interface system. BALF cell differential was assessed using May-Grunwald-Giemsa stained cytospin slides with >1000 cells counted in each specimen to achieve the required 0.1% resolution. (**b**) Percentage of BALF neutrophils and eosinophils (expressed as a fraction of non-epithelial cells) in asthma patients and normal donors (inset). Asthma patients were classified into four inflammatory phenotypes based on the upper limits reported for healthy individuals: E – eosinophilic (>2% eosinophils), N – neutrophilic (>3% neutrophils), Mix – mixed (both criteria fulfilled) and P – paucigranulocytic (inflammatory cells within normal range) as previously described.[2–4] (**c**) Graph shows the frequency of lower airway inflammatory phenotypes in asthma patients. Most patients had either eosinophilia (34%) or neutrophilia (25%) in the lower airways, with only a few (n=3, 9%) showing a mixed phenotype. The remaining patients (31%) had a paucigranulocytic phenotype, i.e., did not have increased numbers of inflammatory cells in BALF.

Figure S2

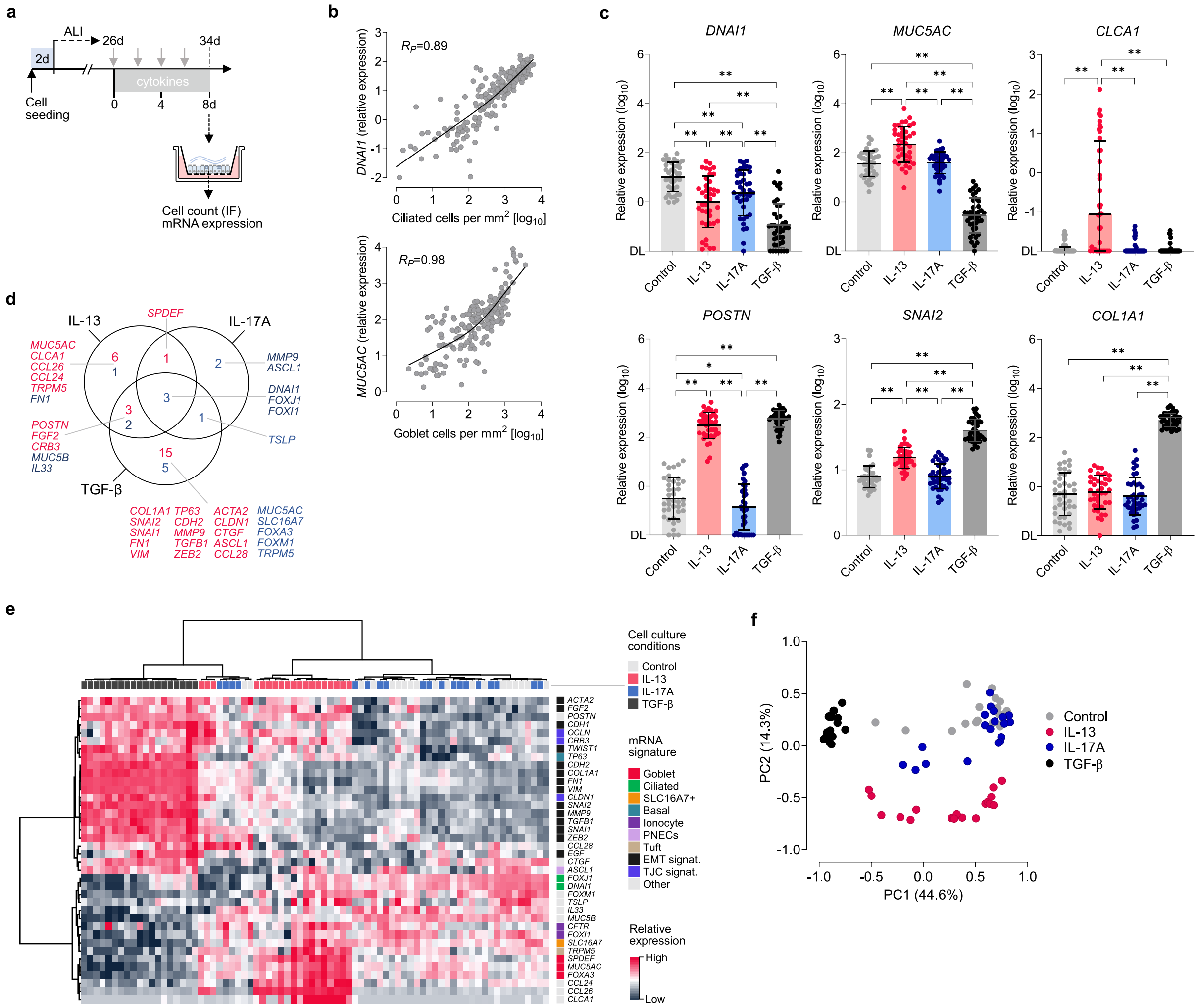

Figure S2 legend. *In vitro* model of cytokine-induced changes in bronchial epithelium. (**a**) Bronchial epithelial cells were initially grown submerged (2 days) followed by air-liquid interface conditions (ALI, 26 days). ALI-epithelia were next cultured in the presence of IL-13, IL-17A, or TGF-β_1 (or control) for an additional 8 days. (**b**) Marked correlation (R_P - Pearson's coefficient) between the number of ciliated or goblet cells and their respective mRNA markers (*DNAI1* or *MUC5AC*). All available data and time points (control, IL-13, and IL-17A conditions) were combined. Data trends were highlighted with spline lines (3-knot). (**c**) Relative expression of *DNAI1*, *MUC5AC*, *CLCA1*, *SNAI2*, *POSTN*, and *COL1A1* in epithelium grown with different cytokines. Bars represent means and SD (n=40, *CLCA1*: median and 0.25-0.75 quartiles). RM 1-way ANOVA (except for *CLCA1*: Friedman test): $*P<0.05$, $**P<0.01$. (**d**) Venn diagram summarizing differences in mRNA expression of epithelial structure and remodeling markers (as listed in panel 'e') in cytokine conditions compared to control. Only targets significantly (n=19, 2-sided paired t-test $P<0.05$, FDRt $q=0.05$) up- (log2fold >1, *red*) or down-regulated (log2fold <1, *navy*) are shown. (**e**) Cluster classification (Ward's) and heat matrix of mRNA expression data (target-centric) in individual ALI-cultures grown in different cytokine conditions (n=19). Color patterns indicate mRNA markers of different airway epithelial cells (e.g., *MUC5AC* as a marker of goblet cells) or processes (e.g., *COL1A1* as a marker of epithelial-mesenchymal transition [EMT]). PNECs – pulmonary neuroendocrine cells, TJC – tight junction complex. We also included *SLC16A7*, a marker of apical (ciliated) cell subset found in the tracheal epithelium with putative involvement in the stress response.[9] (**f**) Principal component (PC) analysis comprising mRNA expression data of structural and remodeling genes confirms the unique gene expression patterns in different culture conditions (n=19).

Figure S3

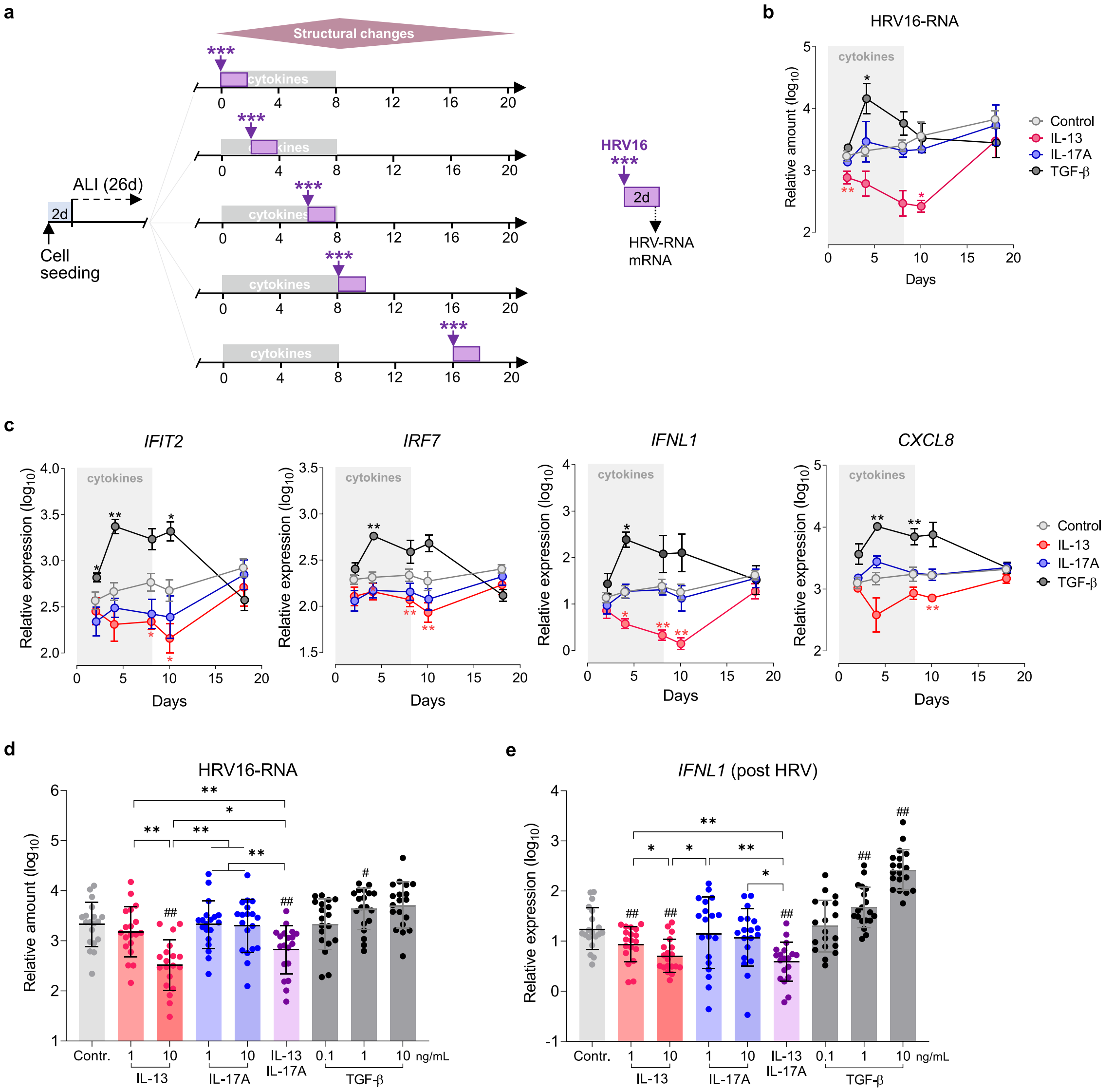

Figure S3 legend. Antiviral response to HRV is related to the intensity of cytokine-induced structural changes of the epithelium. (**a**) ALI-differentiated epithelium (day 26) was cultured in different conditions (8 days: control, IL-13, IL-17A, or TGF-β). To analyze whether the magnitude of structural changes influences the response to HRV, cells were infected with HRV16 (48 h) at different times since the initiation of cytokine treatment (days 0, 2 and 6) and after cytokine withdrawal (days 8 and 16). (**b, c**) Graphs showing a steady decline in the rate of HRV16 replication (HRV16-RNA) and IFN response in IL-13 treated cell cultures (expression of *IFIT2*, *IRF7*, *IFNL1*, and *CXCL8* shown), which was most pronounced at day 8, and shortly after weaning of cytokines. Extended culture of the epithelium in normal conditions (i.e., without cytokines) restored the mucociliary phenotype and resulted in higher sensitivity to HRV infection. This result shows that decreased sensitivity to HRV16 in the epithelium with IL-13-induced metaplasia depends primarily on the structural changes. TGF-β-induced changes (epithelial-mesenchymal transition) resulted in a transient increase in sensitivity to HRV16, which peaked between days 4 and 8. However, the pronounced innate antiviral response was observed during the whole period of incubation with TGF-β and remained elevated shortly after cytokine withdrawal. On the other hand, incubation with IL-17A had little effect on both HRV replication and antiviral response. Data in 'b' and 'c' are presented as means and SEM (n=4). RM 2-way ANOVA (Dunnett in comparison to control): $*P<0.05$, $**P<0.01$. (**d**) Response to HRV infection depends on the cytokine dose. ALI-differentiated epithelia were incubated with cytokines (8 d) and next infected with HRV. The graphs show HRV replication and (**e**) *IFNL1* mRNA expression 48 h post-infection. Data are presented as means and SD (n=19). RM 1-way ANOVA (Tukey). For clarity, only statistical differences in comparison to control (#$P<0.05$, ##$P<0.01$), and within IL-13 and IL-17A datasets ($*$) are shown.

Figure S4

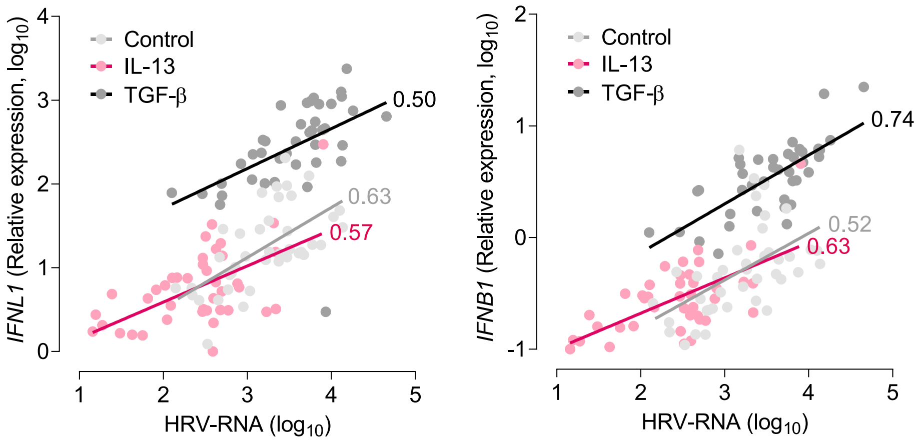

Figure S4 legend. Correlation between virus replication and type I and III IFN mRNA response in HRV16 infected bronchial epithelium. IL-13-induced MCM was characterized by a decrease in HRV16 replication and a parallel decline in IFN response, which overlaid partially with the control dataset. This indicates that the magnitude of innate response to infection is related to the virus load (amount of HRV-RNA in the infected epithelium). In contrast, in epithelium with TGF-β-induced EMT, IFN response was ~10-fold higher even in samples with virus replication rate similar to that observed in other culture conditions. IL-17A data are not shown for clarity. Pearson's R coefficient values are shown next to each regression line.

Figure S5

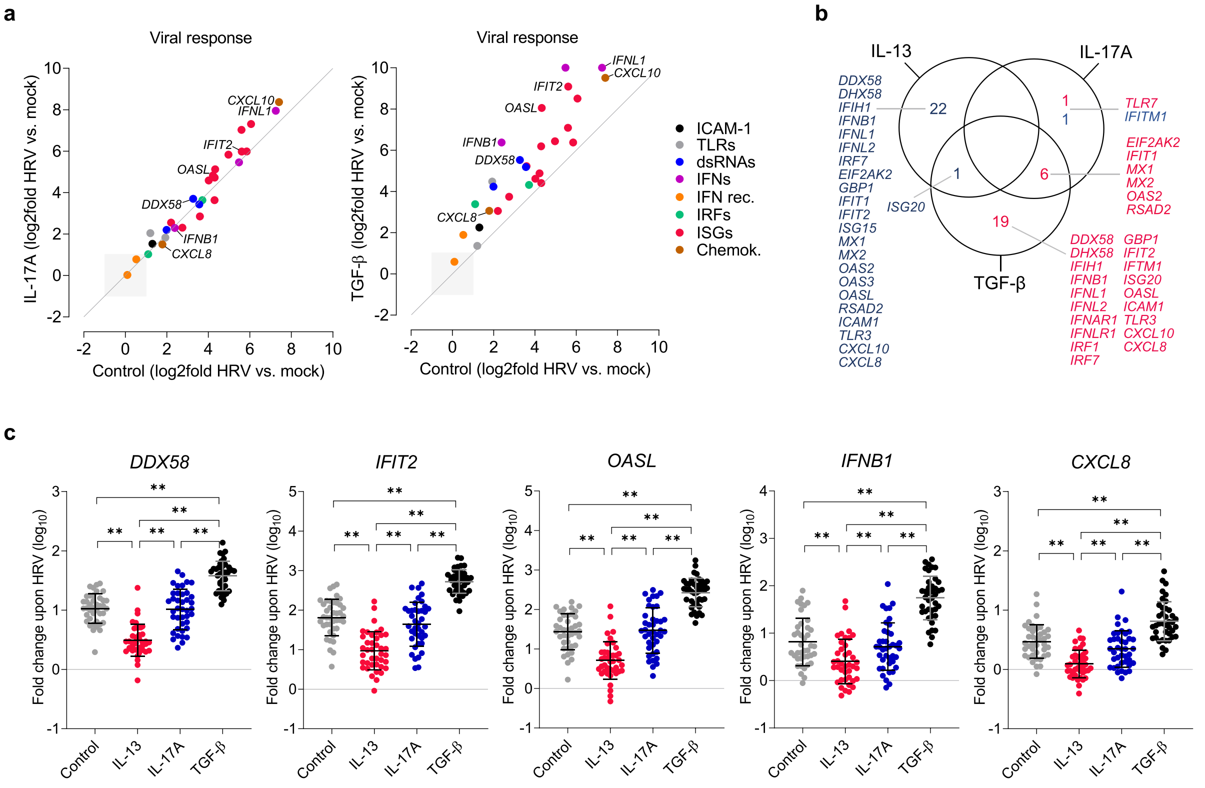

Figure S5 legend. The dynamics of the expression of viral response genes in HRV16 infected epithelium depends on the cytokine treatment. (**a**) *In vitro* differentiated bronchial epithelium was grown in the presence of cytokines and next infected with HRV16. The plots compare fold changes (log2 scale) in the expression of viral response genes upon HRV16 (compared to mock-treated) in different culture conditions (n=19): control vs. IL-17A on the left, control vs. TGF-β on the right. IL-13 data are presented in Fig. 1 in the main body of the manuscript. (**b**) Plot summarizing statistical differences (RM 1-way ANOVA, Tukey: P<0.05) between HRV-related changes of gene expression (as shown in 'a'). Decreased responses were shown almost exclusively in IL-13 treated cultures (*navy* symbols), while increased responses in TGF-β (EMT) conditions (*red*). There was also a tendency to a higher response of ISGs during IL-17A stimulation (log2fold difference significantly higher for: *EIF2AK2*, *IFIT1*, *MX1*, *MX2*, *OAS2* and *RSAD2*). (**c**) Fold change in the expression of selected viral response genes upon HRV16 infection (compared to mock) in different cytokine conditions. Bars represent means and SD (n=40). RM 1-way ANOVA (Tukey): $**P$<0.01.

Figure S6

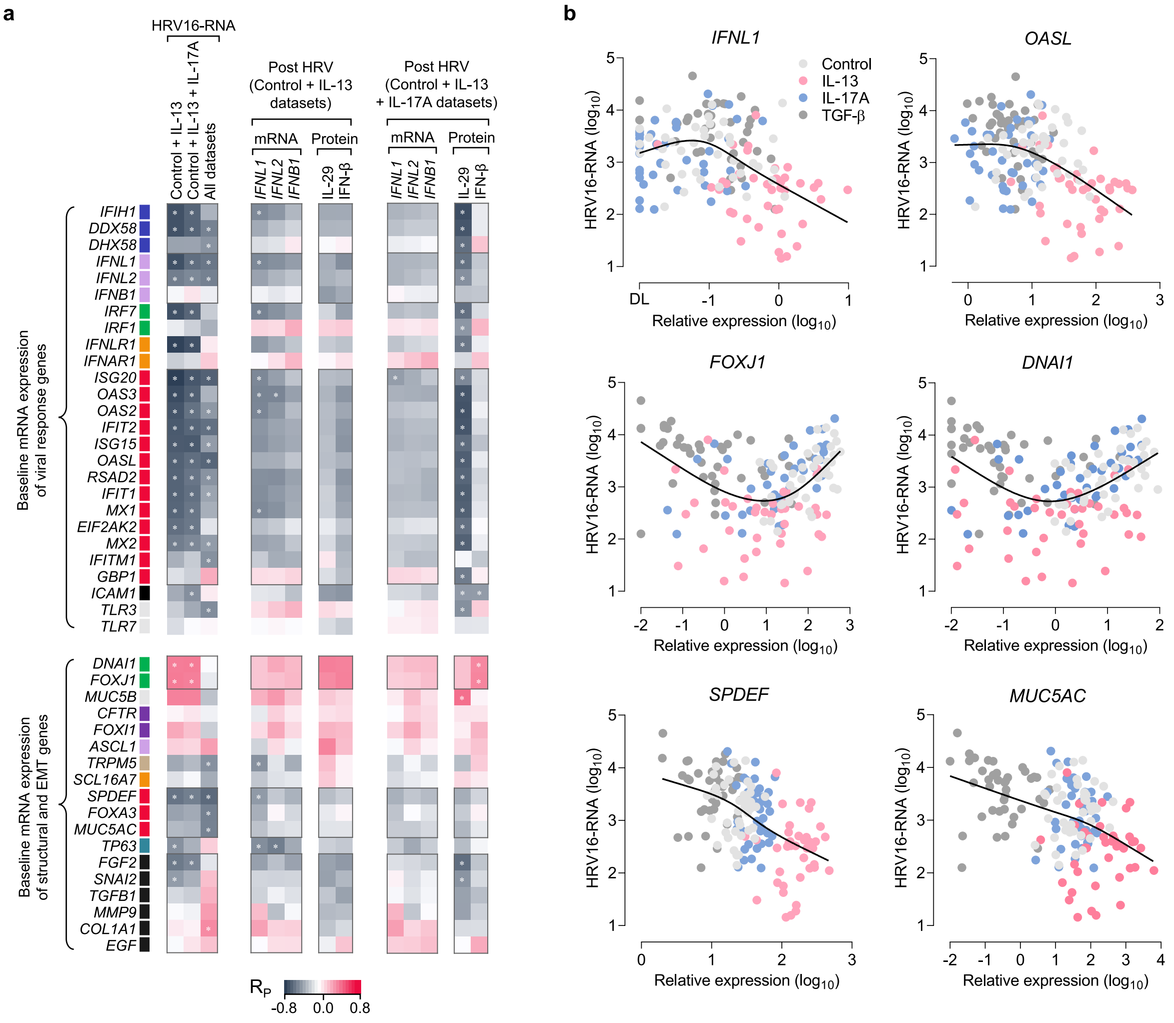

Figure S6 legend. Higher baseline expression of viral response genes is associated with reduced sensitivity to HRV16. (**a**) Correlation heat matrix (Pearson's coefficients [R_P]; culture conditions as indicated) showing linkage between baseline mRNA expression of viral response genes (upper part, colors mark functional groups similarly to Supplementary Fig. S5a, e.g., *red* – IFN-stimulated genes) or selected structural genes (lower part, color legend as in Supplementary Fig. S2e, e.g., *red* – goblet cell signature), and subsequent response to HRV16 infection (HRV16-RNA and type III IFNs). n=19, $*P<0.01$. (**b**) Association between baseline expression of *IFNL1*, *OASL*, *FOXJ1*, *DNAI1*, *SPDEF*, and *MUC5AC* and HRV16 replication (48 h post-infection). Smoothing spline lines (4-knot) are shown to highlight data trends (n=40).

Figure S7

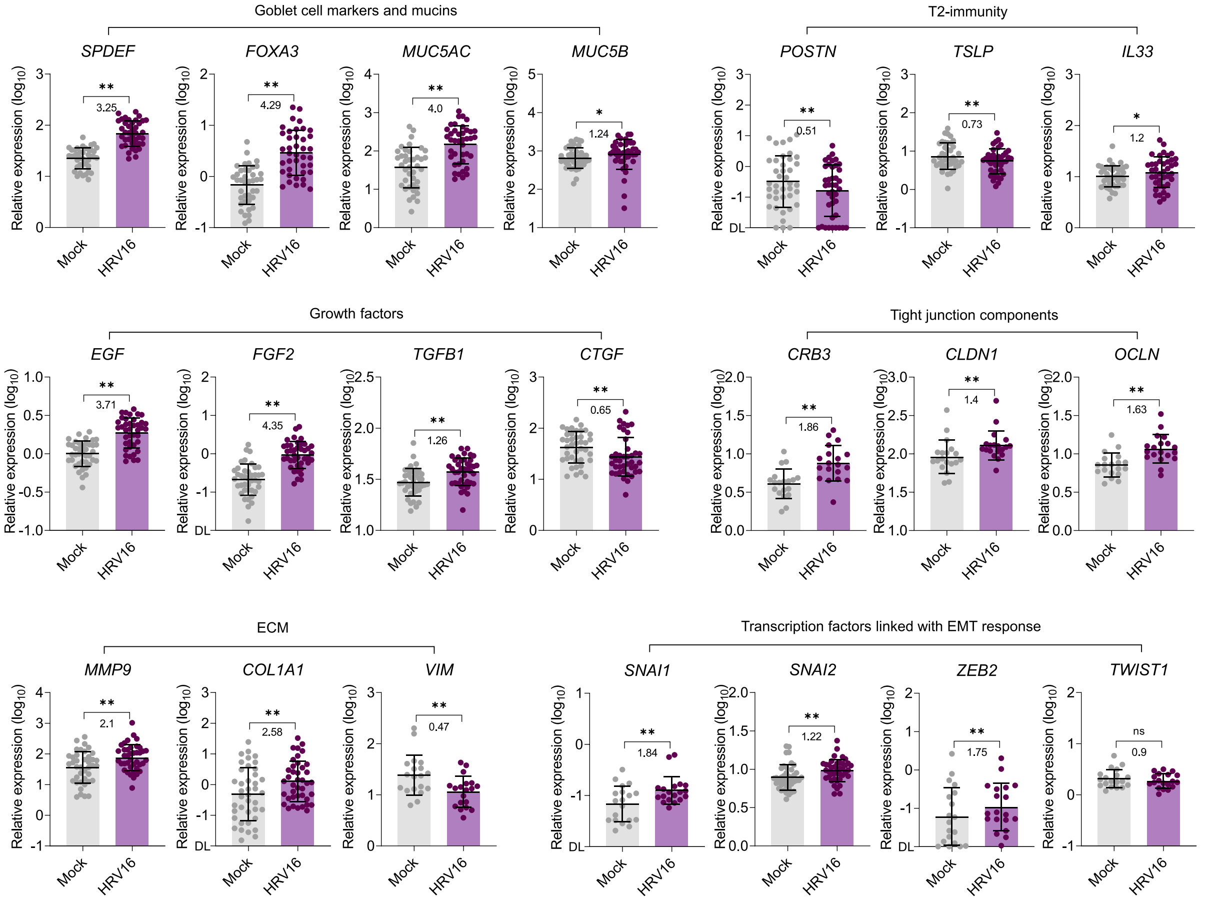

Figure S7 legend. Relative mRNA expression of genes (linked to epithelial structure and remodeling response) in mock or HRV16 infected mucociliary epithelium. Data are shown as means and SD (n=40 or 19). 2-sided paired t-test: ∗P<0.05, ∗∗P<0.01. Numeric values below the significance symbols indicate fold change in the comparison of HRV16 and mock conditions (e.g., 3.25-fold increase in the expression of *SPDEF*, *P*<0.01). ECM – extracellular matrix, EMT – epithelial-mesenchymal transition.

Figure S8

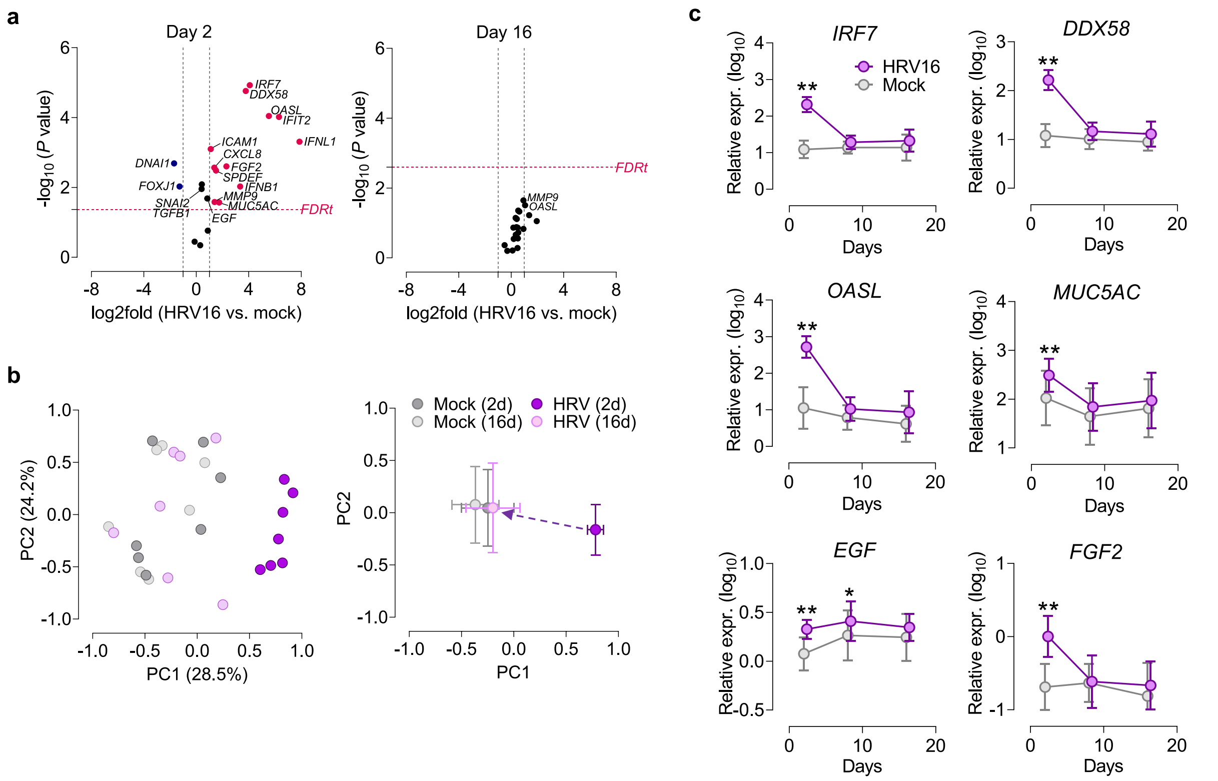

Figure S8 legend. mRNA expression profiles during prolonged HRV16 infection of ALI-grown bronchial epithelium. (**b**) Relative mRNA expression change in mucociliary epithelium 2 d or 16 d post HRV16 infection (gene targets analyzed: *ICAM1, DDX58, IRF7, IFNB1, IFNL1, IFIT2, OASL, CXCL8, DNAI1, FOXJ1, MUC5B, MUC5AC, SPDEF, TSLP, SNAI2, COL1A1, MMP9, EGF, FGF2,* and *TGFB1*). Colors mark genes significantly (n=7; 2-sided t-test *P*<0.05 at the false discovery rate threshold [FDRt] *q*=0.05) upregulated (log2fold >1, *red*) or downregulated (log2fold <-1, *navy*) in comparison to mock. (**b**) Principal component (PC) analysis (all listed genes included) at day 2 and day 16. Graph with mean PC values (whiskers indicate 95CI range) shows the exact overlap between the mRNA profiles on day 16 in both HRV16 infected and mock-treated epithelium. (**c**) Dynamics of mRNA expression (selected genes shown) during prolonged HRV16 infection. Data presented as means and SD (n=7). RM 2-way ANOVA (Sidak): ∗*P*<0.05, ∗∗*P*<0.01 in comparison to mock.

Figure S9

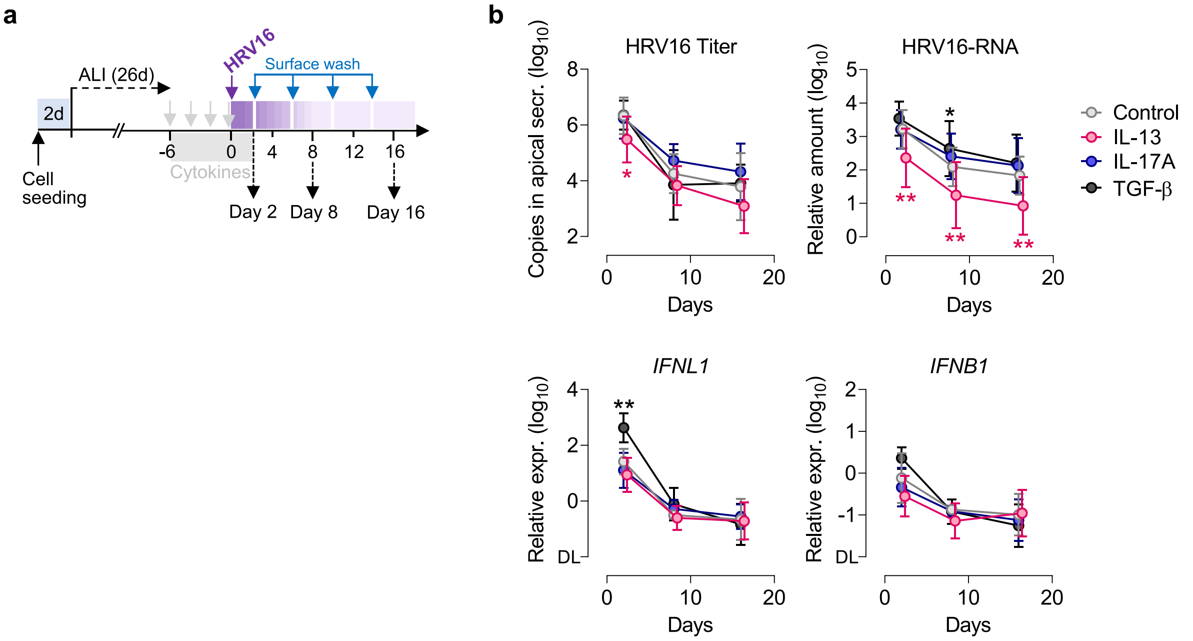

Figure S9 Legend. Cytokine-induced epithelial remodeling does not prolong HRV16 infection and virus shedding. (**a**) Air-liquid interface (ALI) cultures of bronchial epithelium were incubated with IL-13, IL-17A, or TGF-β (8 days) and infected with HRV16. The surface of the epithelium was occasionally washed to mimic mucociliary clearance. Cells were grown without cytokines since day 2 (as indicated). (**b**) The number of HRV16 genome copies detected in the apical supernatant, HRV16-RNA in cell lysates and mRNA expression of *IFNL1* and *IFNB1* in different culture conditions. Data are presented as means and SD (n=7). RM 2-way ANOVA (Sidak), only significant differences in comparison to the control dataset are shown: $*P<0.05$, $**P<0.01$.

Figure S10

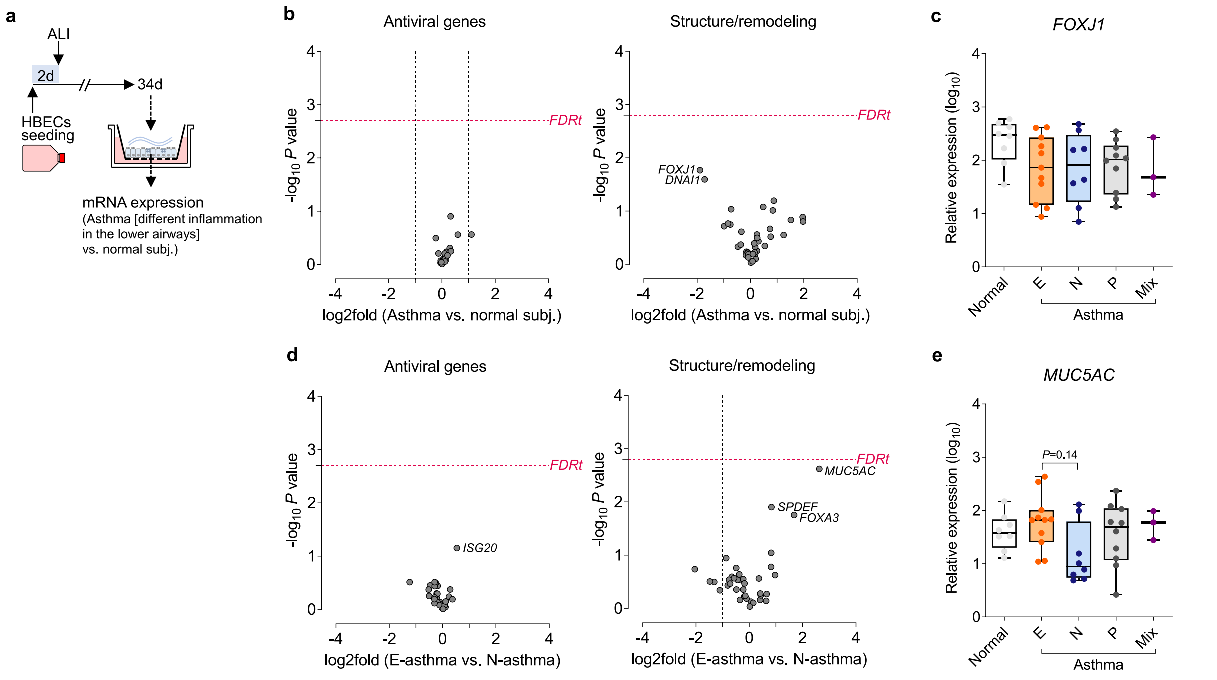

Figure S10 legend. Minor differences in mRNA expression of *in vitro* grown bronchial epithelium derived from asthma patients and control subjects. (**a**) We analyzed mRNA expression (antiviral genes and structural/remodeling genes as listed in Table E2) in bronchial epithelial cells differentiated 34 days in air-liquid interface (ALI)-system. (**b**) Volcano plots comparing mRNA expression in asthma patients (n=15) and normal subjects (n=8). There was no difference in comparison of the two groups, except for a trend toward downregulation of ciliogenesis markers in ALI-grown epithelium from asthma patients (e.g., *FOXJ1* *P*=0.014). (**c**) mRNA expression of *FOXJ1* was similar in asthma patients with different inflammatory phenotypes. (**d**) Relative gene expression differences in ALI-grown epithelium from asthma patients with eosinophilic (E) and neutrophilic (N) inflammation. We observed a trend toward higher expression of mucous cell markers (e.g., *MUC5AC* *P*=0.002, *SPDEF* *P*=0.012) in epithelial cultures derived from patients with E-asthma. (**e**) A similar trend was observed in the validation comprising all asthma patients studied (n=32). Statistics in ‘b’ and ‘d’: Horizontal lines indicate the threshold of significance (n=23; 2-sided t-test *P*<0.05 at the false discovery rate threshold [FDRt] q=0.05). Statistics in ‘c’ and ‘e’: Data are shown as medians and range (n=40; Kruskal-Wallis test, Dunn’s post hoc [Mix dataset excluded due to low sample size]). P – paucigranulocytic asthma, Mix – mixed inflammatory asthma.

Figure S11

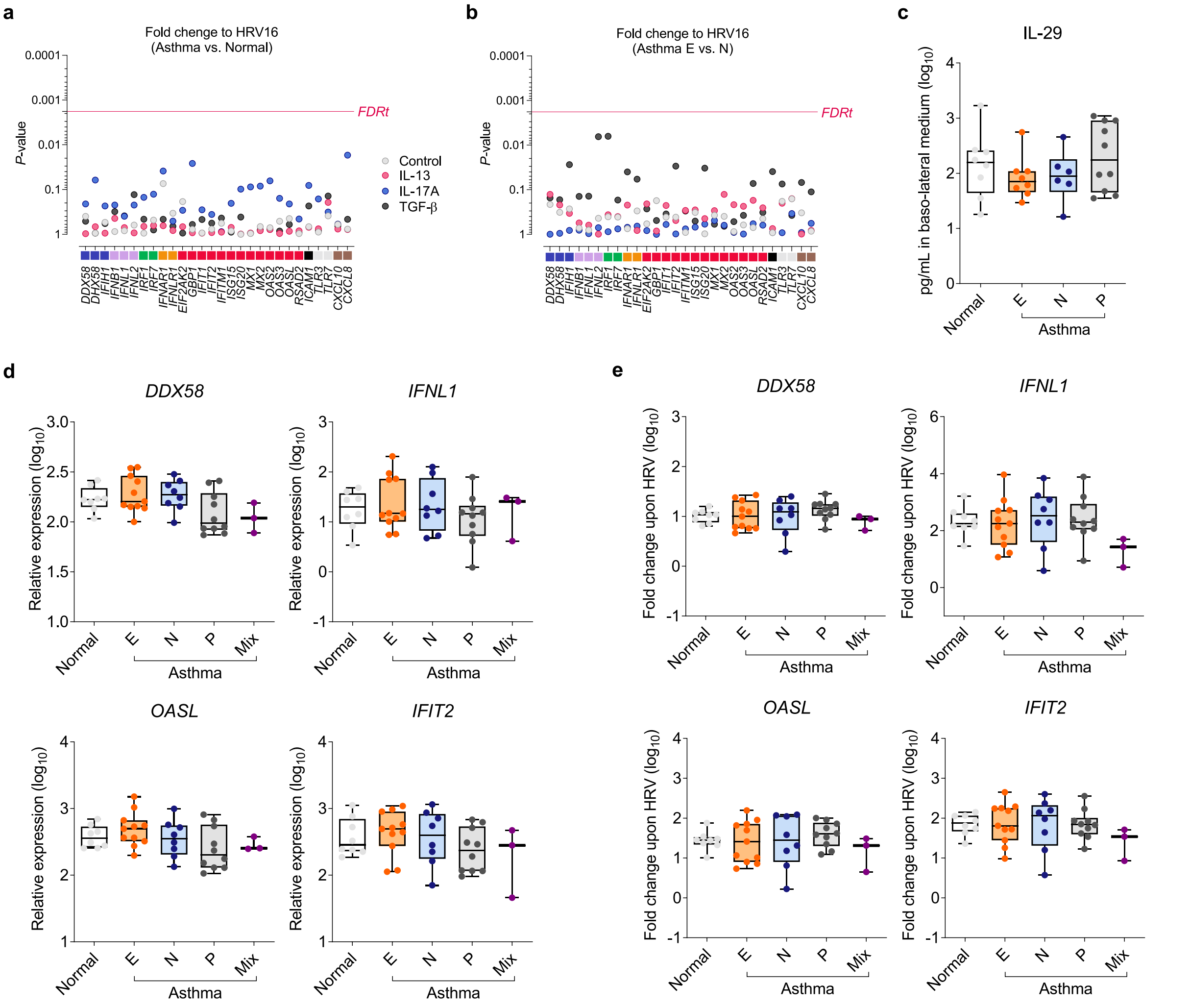

Figure S11 legend. No difference in bronchial epithelial response to HRV16 in comparison of asthma patients with different lower airway inflammation. (**a**) Summary plots showing no difference in HRV-related changes in gene expression in the comparison of asthma patients and normal subjects, and (**b**) asthma patients with eosinophilic (E) or neutrophilic (N) lower airway inflammatory phenotypes, irrespectively of culture conditions (n=19, 2-sided t-test at false discovery rate threshold [FDRt] q=0.05 calculated using Benjamini-Hochberg method). (**c**) Levels of IL-29 (*IFNL1*) in the baso-lateral medium collected 48 h post-HRV16 infection in bronchial epithelial cells derived from asthma patients with different lower airway inflammation (n=32, no difference with Kruskal-Wallis test). (**d**) Similar expression of viral response genes and (**e**) fold changes in response to HRV in ALI-differentiated epithelium from asthma patients with different lower airway inflammation (n=40; no significant between-group difference with Kruskal-Wallis test). P – paucigranulocytic, Mix – mixed inflammatory phenotype.

Figure S12

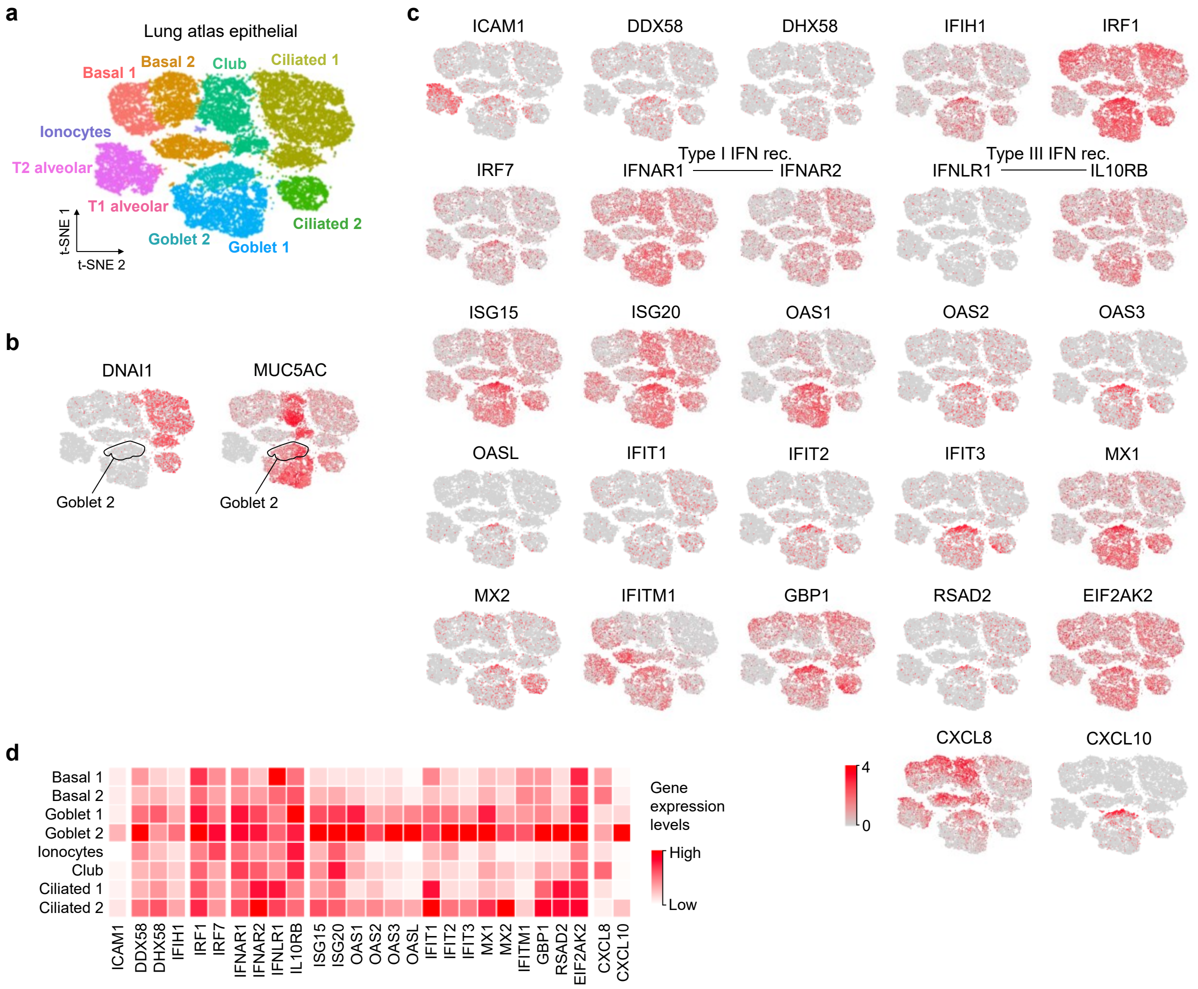

Figure S12 legend. Expression of viral response genes in different airway epithelial cells based on single-cell transcriptome database ‘Lung Cell Atlas’. Using single-cell sequencing, Vieira Braga et al.[10] profiled airway epithelial cells (and lung parenchyma) in biopsies sampled at different respiratory tract levels in healthy donors and asthma subjects. Their data can be explored on-line at https://asthma.cellgeni.sanger.ac.uk. Using this remarkable resource, we were able to track the expression of viral response genes in different subsets of airway epithelial cells (accessed Feb. 02, 2021). (**a**) tSNE plot displaying major airway epithelial cell clusters as displayed in Lung Cell Atlas (epithelial dataset [normal donors], data from different parts of the respiratory tract combined); T1 – type 1, T2 – type 2. Lower airway goblet cells are enriched in the cluster Goblet 2, and lower airway ciliated cells are located mostly in the cluster Ciliated 1. (**b**) tSNE as in ‘a’ showing expression of *DNAI1* (ciliated cells) and *MUC5AC* (enriched within secretory cell clusters). (**c**) tSNE plots (as in ‘a’) highlighting the distribution of cells expressing viral response pathway genes (type I and III interferons were not detected). For example, cells expressing *DDX58*, *IFIH1*, *IRF1*, and most of ISGs were enriched in goblet cell clusters. Interestingly, several ISGs transcripts, such as *ISG15*, *OAS1*, *OAS3*, *OASL*, *IFIT2*, *IFIT3*, and *MX1* were enriched within Goblet 2 cluster. On the other hand, Ciliated 1 cells (lower airway ciliated cells) showed decreased expression of dsRNA sensors and most of ISGs. (d) Average gene expression levels per cell cluster. Heat map data were combined based on individual gene cards available at Lung Cell Atlas website. Color intensity reflects mRNA expression of mRNA-positive cells (target centric, only ‘epithelial’ dataset shown).

Figure S13

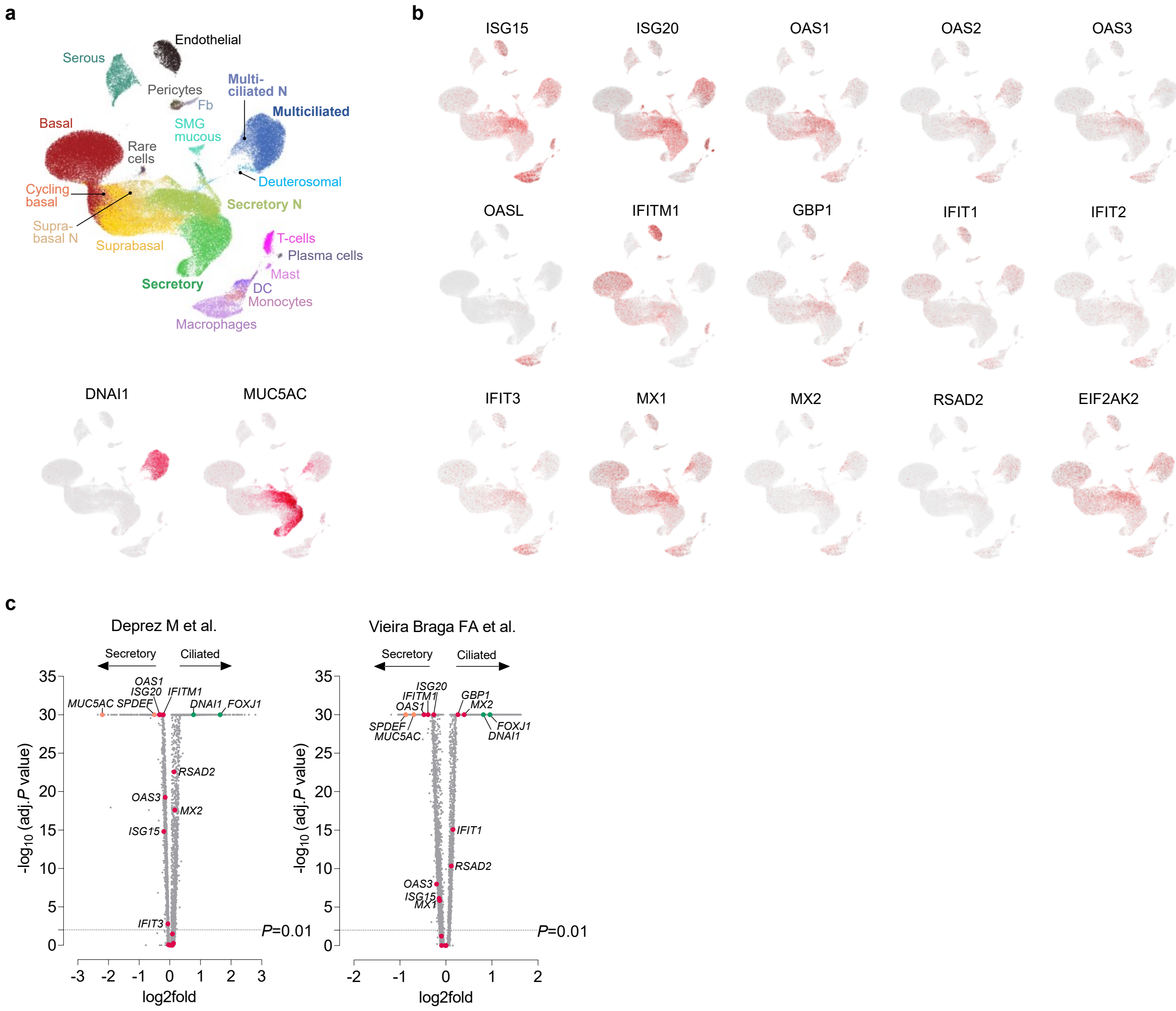

Figure S13 legend. Expression of interferon-stimulated genes (ISGs) in different subsets of airway cells based on online data published by Deprez M et al.[11] and Singh A et al.[12] (**a**) Deprez et al.[11] performed single-cell RNA profiling in different locations of upper and lower airways in 10 healthy individuals. Their data can be accessed via an interactive online tool at https://www.genomique.eu/cellbrowser/HCA/. The graph shows UMAP projection of the whole dataset (hg19 annotation) as presented in UCSC cell browser (accessed Feb. 08, 2021), with major cell clusters highlighted. Abbr.: Fb – fibroblasts, SMG – submucosal gland, DC – dendritic cells, N – cell clusters detected only in nasal samples. Below we show the expression of *DNAI1* and *MUC5AC* enriched in ciliated and secretory cell clusters, respectively. (**b**) UMAP plots with highlighted cells expressing individual ISGs. *ISG15*, *ISG20*, and OAS-family transcripts were enriched in secretory cell subsets. (**c**) In their recent study, Singh A et al.[12] reanalyzed single-cell transcriptome databases in search for the expression of SARS-CoV2 entry factors. Among others, they compared the transcriptome of airway ciliated and secretory (goblet) cells based on data extracted from three studies (Ref.[10,11,13]). Using data from Supplementary Table S7 in Singh A et al.[12] (available at https://ars.els-cdn.com/content/image/1-s2.0-S2211124720311645-mmc8.xlsx) we checked for differences in ISGs expression in comparison of the two cell types. Results from Ruiz Garcia S et al.[13] study are not shown as they covered only nasal epithelial cells. Log2fold values are plotted against adjusted -$\log_{10}$P-values (trimmed at P=10^{-30} for clarity). ISGs (listed in 'b') are marked *red*. Additionally, we highlighted representative transcripts linked to ciliated (*DNAI1*, *FOXJ1*) and secretory (*MUC5AC*, *SPDEF*) signatures. *ISG15*, *ISG20*, and selected OAS-family genes (but not *MX2* and *RSAD2*) were enriched in airway epithelial secretory cells.

4. References

1. Global Initiative for Asthma. Global Strategy for Asthma Management and Prevention, 2020. https://ginasthma.org/.
2. Balbi, B. *et al.* Bronchoalveolar lavage, sputum and exhaled clinically relevant inflammatory markers: Values in healthy adults. *Eur. Respir. J.* **30**, 769–781 (2007).
3. Heron, M. *et al.* Bronchoalveolar lavage cell pattern from healthy human lung. *Clin. Exp. Immunol.* **167**, 523–531 (2012).
4. Jakiela, B. *et al.* Heterogeneity of lower airway inflammation in patients with NSAID-exacerbated respiratory disease. *J. Allergy Clin. Immunol.* **147**, 1–13 (2020).
5. De Groot, J. C., Brinke, A. Ten & Bel, E. H. D. Management of the patient with eosinophilic asthma: A new era begins. *ERJ Open Res.* **1**, 1–11 (2015).
6. Hasegawa, K., Stoll, S. J., Ahn, J., Bittner, J. C. & Camargo, C. A. Prevalence of eosinophilia in hospitalized patients with asthma exacerbation. *Respir. Med.* **109**, 1230–1232 (2015).
7. Simpson, J. L., Scott, R., Boyle, M. J. & Gibson, P. G. Inflammatory subtypes in asthma: Assessment and identification using induced sputum. *Respirology* **11**, 54–61 (2006).
8. Jakiela, B. *et al.* Th2-type cytokine-induced mucus metaplasia decreases susceptibility of human bronchial epithelium to rhinovirus infection. *Am. J. Respir. Cell Mol. Biol.* **51**, 229–241 (2014).
9. Plasschaert, L. W. *et al.* A single-cell atlas of the airway epithelium reveals the CFTR-rich pulmonary ionocyte. *Nature* **560**, 377–381 (2018).
10. Vieira Braga, F. A. *et al.* A cellular census of human lungs identifies novel cell states in health and in asthma. *Nat. Med.* **25**, 1153–1163 (2019).
11. Deprez, M. *et al.* A single-cell atlas of the human healthy airways. *Am. J. Respir. Crit. Care Med.* **202**, 1636–1645 (2020).
12. Singh, M., Bansal, V. & Feschotte, C. A Single-Cell RNA Expression Map of Human Coronavirus Entry Factors. *Cell Rep.* **32**, (2020).
13. Garcıá, S. R. *et al.* Novel dynamics of human mucociliary differentiation revealed by single-cell RNA sequencing of nasal epithelial cultures. *Dev.* **146**, dev177428 (2019).
